# Morphological parameters affecting false lumen thrombosis following type B aortic dissection: a systematic study based on simulations of idealized models

**DOI:** 10.1007/s10237-023-01687-5

**Published:** 2023-01-11

**Authors:** Alireza Jafarinia, Gian Marco Melito, Thomas Stephan Müller, Malte Rolf-Pissarczyk, Gerhard A. Holzapfel, Günter Brenn, Katrin Ellermann, Thomas Hochrainer

**Affiliations:** 1 Institute of Strength of Materials, Graz University of Technology, Graz, Austria; 2 Institute of Mechanics, Graz University of Technology, Graz, Austria; 3 Institute of Fluid Mechanics and Heat Transfer, Graz University of Technology, Graz, Austria; 4 Institute of Biomechanics, Graz University of Technology, Graz, Austria; 5 Department of Structural Engineering, Norwegian University of Science and Technology (NTNU), Trondheim, Norway

**Keywords:** Aortic dissection, Thrombus formation, Global sensitivity analysis, Morphological parametrization, Thrombus classification

## Abstract

Type B aortic dissection (TBAD) carries a high risk of complications, particularly with a partially thrombosed or patent false lumen (FL). Therefore, uncovering the risk factors leading to FL thrombosis is crucial to identify high-risk patients. Although studies have shown that morphological parameters of the dissected aorta are related to FL thrombosis, often conflicting results have been reported. We show that recent models of thrombus evolution in combination with sensitivity analysis methods can provide valuable insights into how combinations of morphological parameters affect the prospect of FL thrombosis. Based on clinical data, an idealized geometry of a TBAD is generated and parameterized. After implementing the thrombus model in computational fluid dynamics simulations, a global sensitivity analysis for selected morphological parameters is performed. We then introduce dimensionless morphological parameters to scale the results to individual patients. The sensitivity analysis demonstrates that the most sensitive parameters influencing FL thrombosis are the FL diameter and the size and location of intimal tears. A higher risk of partial thrombosis is observed when the FL diameter is larger than the true lumen diameter. Reducing the ratio of the distal to proximal tear size increases the risk of FL patency. In summary, these parameters play a dominant role in classifying morphologies into patent, partially thrombosed, and fully thrombosed FL. In this study, we point out the predictive role of morphological parameters for FL thrombosis in TBAD and show that the results are in good agreement with available clinical studies.

**Supplementary Information:**

The online version contains supplementary material available at 10.1007/s10237-023-01687-5.

## Introduction

Type B aortic dissection (TBAD) is a relatively rare medical condition with a high mortality rate in which a tear in the intimal layer of the descending part of the aorta allows blood to flow between the layers of the aortic wall, causing them to separate. This creates a false lumen (FL) that originates in the descending aorta and may expand into the abdomen as the disease progresses (LeMaire and Russell 2011). In most cases, one or more ‘re-entry’ tears (Erbel et al. 2001) form an additional path for blood flow between the true lumen (TL) and the FL (Nienaber et al. 2016). Since TBAD is associated with late adverse events such as aortic rupture, rapid aortic growth ($>{10}$ mm/yr), aneurysm formation (aortic diameter $\ge {6}$ cm), and organic and limb ischemia (Sailer et al. 2017), it requires regular medical monitoring, which remains a challenge despite recent advances in clinical practice (Rudenick et al. 2017).

A significant predictor for late dissection-related deaths and re-treatment of the descending aorta is the FL status, which refers to the amount of thrombus in the FL (Evangelista et al. 2012). Most commonly, the FL status is classified as patent (thrombus free), partially thrombosed, or completely thrombosed, whereby the FL growth and the mortality rates of TBAD patients are usually found to be significantly higher in the case of a patent or a partially thrombosed FL (Bernard et al. 2001; Akutsu et al. 2004; Sueyoshi et al. 2004; Trimarchi et al. 2006; Tsai et al. 2007; Fattouch et al. 2009; Trimarchi et al. 2013). The fastest aortic growth and the highest mortality rates are frequently associated with a partially thrombosed FL. For instance, compared to patients with a patent FL, Tsai et al. (2007) reported that the risk of death for patients with a partially thrombosed FL is higher by a factor of 2.7. However, we note that there are a few studies (Sueyoshi et al. 2009; Jonker et al. 2012) that do not support the finding that a partially thrombosed FL would be a predictor of late adverse events. More unequivocally, a complete FL thrombosis is found to improve the outcomes of patients with TBAD (Bernard et al. 2001; Akutsu et al. 2004; Sueyoshi et al. 2004; Trimarchi et al. 2006; Tsai et al. 2007; Fattouch et al. 2009; Trimarchi et al. 2013; Tanaka et al. 2014; Naim et al. 2016); in particular, it decreases the risk of death and it is associated with the lowest FL growth rates. A complete thrombosis in the FL can slow down or potentially stop the dissection progression.

**Fig. 1:**
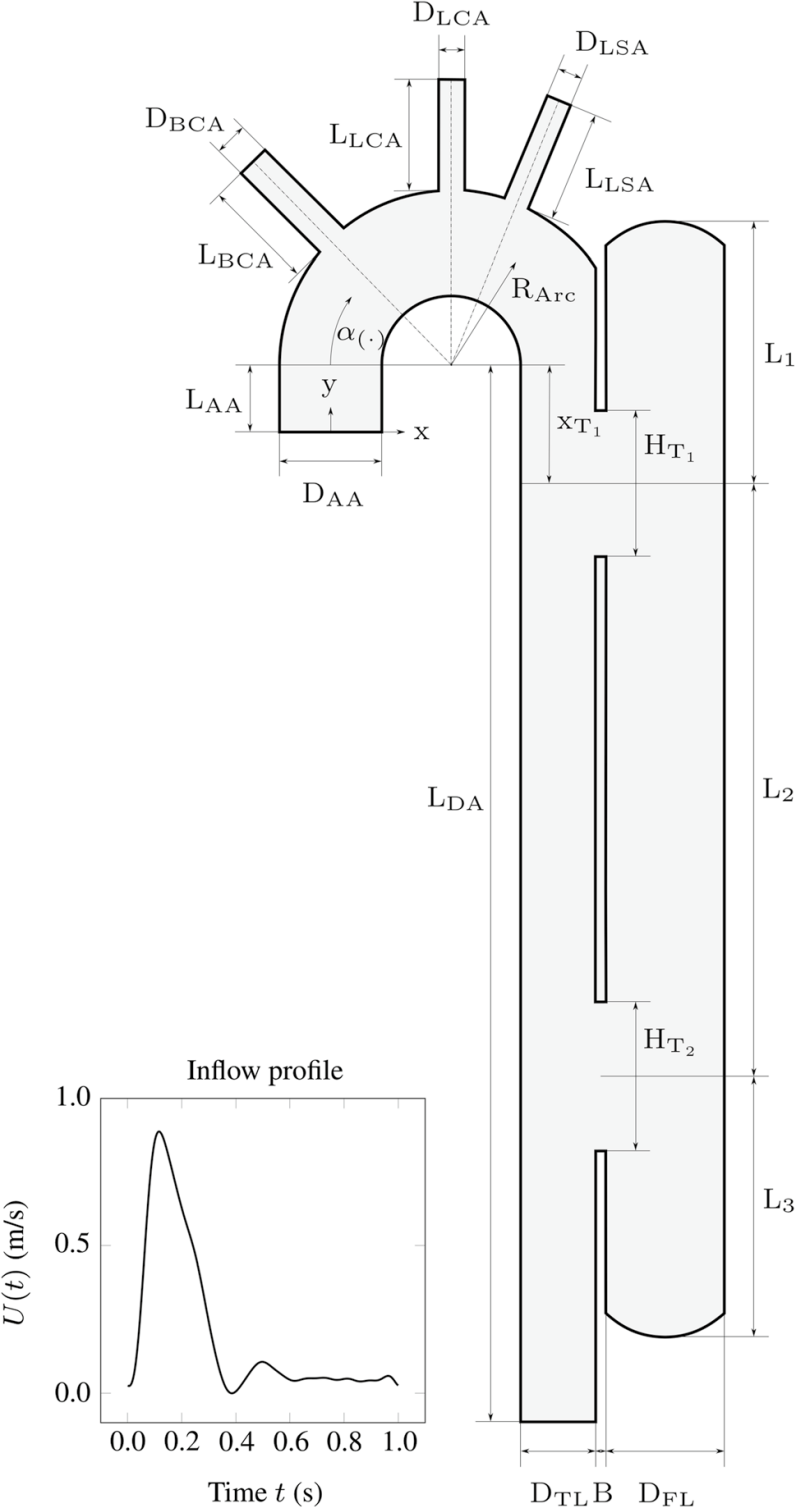
An idealized two-dimensional geometry of the TBAD morphology and definition of the morphological parameters and variables, with the transient velocity profile imposed at the inlet over one cardiac cycle ($T={1}$ s). The angle $\alpha _{(\cdot )}$ defines the position of the BCA ($\alpha _\mathrm{BCA}$), the LCA ($\alpha _\mathrm{LCA}$) and the LSA ($\alpha _\mathrm{LSA}$)

From a clinical point of view, the classification of patients into various risk groups with respect to the likelihood of FL thrombosis is desirable. Thrombosis is known to be governed by hemodynamic conditions. In a series of experimental studies, correlations between hemodynamics and morphological parameters were investigated (Mohr-Kahaly et al. 1989; Iwai et al. 1991; Chung et al. 2000a, b; Tsai et al. 2008; Qing et al. 2012; Rudenick et al. 2013; Canchi et al. 2018). The hemodynamics is clearly influenced by the aortic morphology in terms of location, size, and the number of intimal tears and also the dimensions of the FL. Therefore, it seems that the geometry of the dissected aorta might be used for classifying patients, but studies related to the direct correlation of morphology to thrombus formation are rare or even not available (Pepe et al. 2020).

Solely one combined experimental and computational study was performed by Zadrazil et al. (2020) to determine the flow characteristics in the lumina with four different tear sizes by using idealized morphologies of the dissected aorta. As shown by their experiments, an increase in the distal tear size enhances the FL flow rate and decreases the wall shear stress. In contrast to that, a larger proximal tear size increases both. The highest wall shear stress was located at the edge of the tear. Although they could not quantify the actual influence of these morphological parameters, the study demonstrated that the size of the tears affects FL thrombosis.

**Table 1:** Values of (constant) morphological parameters of the idealized geometry (Erbel 2006; Evangelista et al. 2012; Vasava et al. 2012; Craiem et al. 2016; Menichini and Xu 2016)

Parameter	Value (mm)	Parameter	Value (mm)	Parameter	Value (deg)
$\mathrm {L}_{\mathrm {AA}}$	18	$\mathrm {D}_{\mathrm {AA}}$	25	$\alpha _\mathrm{BCA}$	45
$\mathrm {L}_{\mathrm {BCA}}$	28	$\mathrm {D}_{\mathrm {BCA}}$	8	$\alpha _\mathrm{LCA}$	90
$\mathrm {L}_{\mathrm {LCA}}$	28	$\mathrm {D}_{\mathrm {LCA}}$	6	$\alpha _\mathrm{LSA}$	112
$\mathrm {L}_{\mathrm {LSA}}$	28	$\mathrm {D}_{\mathrm {LSA}}$	6		
$\mathrm {L}_{\mathrm {DA}}$	250	$\mathrm {B}$	1		
$\mathrm {R}_{\mathrm {Arc}}$	30				

Thrombus growth models in conjunction with fluid dynamics simulations of blood flow have been recently employed for investigating thrombus formation in TBAD, e.g. Menichini and Xu (2016); Menichini et al. (2016, 2018). In these types of simulations, local hemodynamic conditions which promote thrombus formation are for example low wall shear stresses, low shear rates, and high residence times of blood constituents. By employing an idealized geometry of a dissected aorta, Menichini and Xu (2016) found that the distance between proximal and distal tears strongly affected thrombus formation in the FL. Also, in other computational models, the correlation between thrombus growth in the FL and selected morphological parameters was demonstrated (Fan et al. 2010; Ben Ahmed et al. 2016; Naim et al. 2016). However, these studies only concentrated on a few model cases or varying single parameters which makes the ill-suited as a basis for a systematic classification of dissected aorta morphologies. To lay the foundations for such a systematic classification regarding the expected false lumen thrombosis is the aim of the current work.

The value of absolute aortic dimensions for clinical prognosis or intervention has been already questioned in other aortic diseases (Pape et al. 2007; Boudoulas et al. 2018). For instance, regarding aneurysmal dilatation, a fixed value of the aortic diameter of 5.5 cm is normally used for deciding on surgical intervention (Gott et al. 1999). However, we also analyze the results in terms of dimensionless parameters, e.g. relative values of morphological dimensions, like the relative size of FL- to TL-diameter which have been found to be of importance for thrombosis by Spinelli et al. (2018).

In the current study, we consider a variety of morphological parameters of dissected aortas and conduct a comprehensive analysis of their combined influence on thrombus formation. A global sensitivity analysis is also carried out to identify the dominant morphological parameters affecting the thrombus coverage in the FL.

A thrombus formation model was incorporated into computational fluid dynamics (CFD) simulations and a parameterization of morphologies representing TBAD was developed. The simulations are performed in the open-source software *OpenFOAM* (Weller et al. 1998) implementing the combination of a non-Newtonian blood flow behavior (Jafarinia et al. 2020) and the phenomenological hemodynamic-based model for thrombus formation developed by Menichini and Xu (2016). We employ this phenomenological thrombus formation model since detailed modeling of the biochemical interactions between blood species involved in the hemostasis process would be computationally intractable (Menichini and Xu 2016; Chong et al. 2022). Therefore, to speed up the thrombus growth, a hemodynamic-based model which has simplified the thrombosis process is used in the current study. Moreover, in order to enable a comprehensive parameter variation as required by the global sensitivity analysis, the simulations are performed on idealized two-dimensional (2D) TBAD morphologies. These simplified morphologies are able to incorporate important morphological characteristics of the aorta in TBAD, including the aortic arch, various dimensions of TL and FL, and different sizes and locations of the intimal tears. It was shown by Menichini and Xu (2016), who investigated the influence of a few such morphological parameters on thrombus formation, that the results predicted from similar 2D morphologies were in qualitative agreement with clinical studies.

A global sensitivity analysis is applied to the proposed computational model. Due to the complex behavior of the computational model, we employ a variance-based sensitivity analysis in which the output is decomposed into a sum of contributions of the input space (Saltelli et al. 2008). We then examine the percentage of thrombus coverage in the FL as the quantity of interest in the sensitivity analysis. Based on the variation of the thrombus coverage in dependence on the morphological parameters, the sensitivity analysis reveals the most sensitive parameters with regard to thrombus formation. These results are used to classify the morphologies of TBADs based on the prospect of developing a patent, a partially or a completely thrombosed FL. For this classification, we suggest using dimensionless numbers, defined as the relative values between absolute morphological parameters, which allows transferring the results between patients with different absolute aortas sizes and accounting for patients’ individuality.

**Table 2:** Values of (variable) morphological parameters of the idealized geometry and their probability ranges

Description	Absolute parameter	Range (mm)		Reference
		Min	Max	
Proximal FL length	$\mathrm {L}_\mathrm {1}$	4.0	42.5	Bernard et al. (2001); Craiem et al. (2016); Erbel (2006); Evangelista et al. (2012); Karmonik et al. (2011), (2010); Khoynezhad et al. (2010); Quint et al. (2003); Strotzer et al. (2000); Tsai et al. (2008)
Medial FL length	$\mathrm {L}_\mathrm {2}$	8.5	164.0	Bernard et al. (2001); Craiem et al. (2016); Erbel (2006); Evangelista et al. (2012); Karmonik et al. (2011), (2010); Khoynezhad et al. (2010); Quint et al. (2003); Strotzer et al. (2000); Tsai et al. (2008)
Distal FL length	$\mathrm {L}_\mathrm {3}$	4.0	42.5	Bernard et al. (2001); Craiem et al. (2016); Erbel (2006); Evangelista et al. (2012); Karmonik et al. (2011), (2010); Khoynezhad et al. (2010); Quint et al. (2003); Strotzer et al. (2000); Tsai et al. (2008)
FL diameter	$\mathrm {D}_\mathrm {FL}$	6.5	30.0	Bernard et al. (2001)
Proximal tear size	$\mathrm {H}_\mathrm {T1}$	1.4	25.0	Evangelista et al. (2012); Tsai et al. (2008); Quint et al. (2003)
Distal tear size	$\mathrm {H}_\mathrm {T2}$	0.4	33.0	Evangelista et al. (2012); Tsai et al. (2008); Quint et al. (2003)
TL diameter	$\mathrm {D}_\mathrm {TL}$	12.5	25.0	Evangelista et al. (2012); Tsai et al. (2008); Quint et al. (2003); Erbel (2006); Bernard et al. (2001); Craiem et al. (2016)
Proximal tear location	$\mathrm {x}_\mathrm {T1}$	$-36.3$	25.6	Tsai et al. (2008); Khoynezhad et al. (2010); Karmonik et al. (2010), (2011); Evangelista et al. (2012)

**Table 3:** Definition of dimensionless morphological parameters

Dimensionless parameter	Definition	Description
$\mathrm {L}_\mathrm {1}^*$	$\mathrm {L}_\mathrm {1}/\mathrm {D}_\mathrm {FL}$	Proximal slenderness
$\mathrm {L}_\mathrm {2}^*$	$\mathrm {L}_\mathrm {2}/\mathrm {D}_\mathrm {FL}$	Medial slenderness
$\mathrm {L}_\mathrm {3}^*$	$\mathrm {L}_\mathrm {3}/\mathrm {D}_\mathrm {FL}$	Distal slenderness
$\mathrm {D}_\mathrm {FL}^*$	$\mathrm {D}_\mathrm {FL}/\mathrm {D}_\mathrm {TL}$	FL-to-TL diameter ratio
$\mathrm {H}_\mathrm {T1}^*$	$\mathrm {H}_\mathrm {T1}/\mathrm {D}_\mathrm {TL}$	Proximal tear aspect ratio
$\mathrm {H}_\mathrm {T2}^*$	$\mathrm {H}_\mathrm {T2}/\mathrm {H}_\mathrm {T1}$	Distal to proximal tear size ratio
$\mathrm {D}_\mathrm {TL}^*$	$\mathrm {D}_\mathrm {TL}/\mathrm {D}_\mathrm {AA}$	TL to arch diameter ratio
$\mathrm {x}_\mathrm {T1}^*$	$\mathrm {x}_\mathrm {T1}/\mathrm {D}_\mathrm {TL}$	Proximal tear deviation

The investigation of the importance of morphological parameters on FL thrombosis should ultimately contribute to medical diagnostics and patient prognosis, thus helping to accelerate the necessary prevention and therapy planning, especially in high-risk patients.

## Methods

### Parameterization of idealized geometry

An illustration of the idealized two-dimensional (2D) geometry of a TBAD is shown in Fig. 1. The idealized geometry includes the ascending and descending aortas with the TL, the FL, and the carotid arteries, namely the brachiocephalic (BCA), left common carotid (LCA), and left subclavian artery (LSA). To allow blood flow communication between the lumina, the TL and the FL are connected by a proximal and a distal tear.

For this study, we distinguish between two sets of parameters. The first set, which is used for the geometry of the aortic arch, was kept constant, see Table 1. The parameters in the second set are treated as stochastic variables, representing the morphological variation of TBAD. The ranges of these parameters were estimated from clinical and experimental data, as displayed in Table 2. The proximal and distal FL lengths, i.e. $\mathrm {L}_\mathrm {1}$ and $\mathrm {L}_\mathrm {3}$, identify the distance of the center line of the proximal and distal tears from the top and the bottom of the FL, respectively. The medial FL length $\mathrm {L}_\mathrm {2}$ is the distance between the center lines of the two tears. $\mathrm {D}_\mathrm {TL}$ and $\mathrm {D}_\mathrm {FL}$ represent the diameters of the TL and FL. Furthermore, the proximal and distal tear sizes, namely $\mathrm {H}_\mathrm {T1}$ and $\mathrm {H}_\mathrm {T2}$, define the opening dimensions of the intimal tears. Lastly, the proximal tear location $\mathrm {x}_\mathrm {T1}$ identifies the distance between the entry tear center line and the center of the aortic arch; note that $\mathrm {x}_\mathrm {T1}$ is negative for a more distal entry tear. Due to unavailable statistical data on the variation of the morphological parameters, a uniform distribution is assigned to the variables to account for the human variability in the geometrical features of the cardiovascular system. Furthermore, we introduce dimensionless parameters expressed as ratios as shown in Table 3 to make the results applicable to a variety of patient-specific geometries with different absolute dimensions.

### Rheological model and boundary conditions

**Table 4:** Values of model parameters of the rheological and thrombus formation models

Description	Parameter	Value	Unit	Reference
*Rheological model*				
Viscosity at zero shear rate	$\eta _0$	$1.581\times 10^{-2}$	pa s	Jafarinia et al. (2020)
Viscosity at infinite shear rate	$\eta _{\infty }$	$2.779\times 10^{-3}$	pa s	Jafarinia et al. (2020)
Time constant	$\lambda$	1.561	s	Jafarinia et al. (2020)
Power index	$n_\eta$	0.475	–	Jafarinia et al. (2020)
Blood density	$\rho$	$1.060\times 10^{3}$	kg/m$^3$	Jafarinia et al. (2020)
*Thrombus model*				
Bounded platelets concentration threshold	$c_\mathrm {BPt}$	$2\times 10^{4}$	nmol/m$^3$	Menichini and Xu (2016); Menichini et al. (2016)
Coagulant concentration threshold	$c_\mathrm {t}$	$1\times 10^{4}$	nmol/m$^3$	Menichini and Xu (2016)
Residence time threshold	$\langle {T}_{\mathrm {R}}\rangle _\mathrm {t}$	$9\times 10^{-1}$	–	Menichini and Xu (2016)
Shear rate threshold	$\langle \dot{\gamma } \rangle _\mathrm {t}$	50	s$^{-1}$	Menichini et al. (2016)
Coefficient in Navier–Stokes sink term	$k_\mathrm {th}$	$1\times 10^{7}$	kg/m$^3$/s	Menichini and Xu (2016); Menichini et al. (2016)
Reaction rate bounded platelets	$k_\mathrm {BP}$	$8\times 10^{-10}$	nmol/m$^3$/s	Menichini and Xu (2016); Menichini et al. (2016)
Reaction rate coagulant	$k_\mathrm {c}$	$2\times 10^{5}$	nmol/m$^3$/s	Menichini and Xu (2016; Menichini et al. (2016)
Reaction rate, effect of activated platelets on further activation of platelets	$k_1$	$1.2\times 10^{-14}$	m$^3$/s	Menichini and Xu (2016; Menichini et al. (2016)
Reaction rate, effect of exposure of platelets to thrombin	$k_2$	$5\times 10^{-1}$	s$^{-1}$	Menichini and Xu (2016; Menichini et al. (2016)
Brownian diffusion coefficient	$D_\mathrm {b}$	$1.6\times 10^{-13}$	m$^2$/s	Menichini and Xu (2016)
Diffusion coefficient of coagulant	$D_\mathrm {c}$	$10^{-8}$	m$^2$/s	Menichini and Xu (2016; Menichini et al. (2016)
Coagulant reaction rate at wall	$k_\mathrm {cw}$	$2\times 10^{4}$	nmol/m$^2$/s	Menichini et al. (2016)
Shear-enhancing coefficient	$\beta$	$1.6\times 10^{-3}$	m$^2$	Menichini and Xu (2016)
Blood self-diffusion coefficient	$D_{{T}_{\mathrm {R}}}$	$1.14\times 10^{-11}$	m$^2$/s	Menichini and Xu (2016)

The blood is modeled as an incompressible fluid with a constant density $\rho$, such that the mass balance reduces to the solenoidality of the fluid velocity field $\varvec{u}$, i.e. $\nabla \cdot \varvec{u} = 0$. The Navier–Stokes equation for the blood flow reads

1 $$\begin{aligned} \rho \left[ \frac{\partial \varvec{u}}{\partial t} + (\varvec{u} \cdot \nabla )\varvec{u} \right] = -\nabla p + \nabla \cdot \varvec{\tau } - k_\mathrm {th} \phi _{\mathrm {th}} \varvec{u}\,, \end{aligned}$$

with pressure *p* and the extra stress tensor $\varvec{\tau }$. The Navier–Stokes equation is modified by the sink term $k_\mathrm {th} \phi _{\mathrm {th}} \varvec{u}$, which involves a field variable quantifying the degree of local thrombosis $\phi _{\mathrm {th}}$, and a coefficient $k_\mathrm {th}$, which is chosen to stop the flow for $\phi _{\mathrm {th}}=1$. The degree of local thrombosis $\phi _{\mathrm {th}}$ is defined in the range of $0\le \phi _{\mathrm {th}}\le 1$. The value of the coefficient $k_\mathrm {th}$ is taken from Menichini and Xu (2016) such that the sink term stops the blood flow where thrombus is formed, i.e. where $\phi _{\mathrm {th}}\approx 1$.

The degree of local thrombosis $\phi _{\mathrm {th}}$ depends on the concentration of bounded platelets, $c_{\mathrm {BP}}$, through

2 $$\begin{aligned} \phi _{\mathrm {th}}(c_{\mathrm {BP}},c_{\mathrm {BPt}}) = \frac{c_{\mathrm {BP}}^2}{c_{\mathrm {BP}}^2+c_{\mathrm {BPt}}^2}\,, \end{aligned}$$

where $c_{\mathrm {BPt}}$ denotes a threshold value above which the bounded platelets are supposed to clot and form a thrombus. Note that in the following, the subscript notation $\mathrm {t}$ always indicates threshold values of the according field variables.

The employed rheological model considers blood as a shear-thinning liquid with yield stress. The extra stress tensor $\varvec{\tau }$ is given as a function of the rate-of-deformation tensor $\varvec{D}$ and the yield stress $\tau _{\mathrm {y}}$ by

3 $$\begin{aligned} \begin{array}{lll} \varvec{\tau }=2 \eta (\dot{\gamma }) \varvec{D}+\tau _{\mathrm {y}} \frac{1}{\dot{\gamma }} \varvec{D}, &{} \text{ if } &{} |\varvec{\tau }| \ge \tau _{\mathrm {y}}, \\ \varvec{D}=\mathbf {0}, &{} \text{ if } &{} |\varvec{\tau }|<\tau _{\mathrm {y}}\,. \end{array} \end{aligned}$$

The rate-of-deformation tensor $\varvec{D}$ is defined as the symmetric part of the spatial velocity gradient $\nabla \varvec{u}$, i.e.

4 $$\begin{aligned} \varvec{D} = \frac{1}{2} \left( \nabla \varvec{u} + \nabla \varvec{u}^{\mathrm {T}}\right) \,, \end{aligned}$$

and magnitudes of viscous stress and rate of strain are respectively given by

5 $$\begin{aligned} |\varvec{\tau }|&=\sqrt{{\text {tr}}\left( \varvec{\tau }^{2}\right) / 2}\,, \end{aligned}$$

6 $$\begin{aligned} \dot{\gamma }&=\sqrt{2 {\text {tr}}\left( \varvec{D}^{2}\right) }\,. \end{aligned}$$

As for the shear thinning, we employ the Carreau model (Carreau 1968),

7 $$\begin{aligned} \eta ( \dot{\gamma }) = \eta _{\infty } + (\eta _0 - \eta _{\infty }) \Big [1 + (\lambda \dot{\gamma })^2 \Big ]^{(n_\eta -1)/2} \,, \end{aligned}$$

where the rheological model parameters $\eta _0$, $\eta _{\infty }$, $\lambda$, and $n_\eta$ were determined from experimental data in (Jafarinia et al. 2020). The values of the parameters are listed in Table 4.

The inflow velocity is prescribed as a parabolic velocity profile over the inlet cross section as

8 $$\begin{aligned} u_\mathrm {AA}(y,t) = \frac{3}{2}U(t) \left( 1 - \frac{y^2}{\left( \mathrm {D}_{\mathrm {AA}}/2 \right) ^2}\right) \quad \mathrm {at} \quad x=0\,, \end{aligned}$$

where $\mathrm {D}_{\mathrm {AA}}$ is the width of the inlet, which represents the diameter of the ascending aorta in the current 2D geometry. The flow is modeled as fully developed at the inlet so that only the *x*-component of the velocity vector is non-zero. The function *U*(*t*) characterizes the time dependent flow over a heart cycle. This function was adapted from Alastruey et al. (2016) and is shown in Fig. 1. The prescribed cycle-averaged velocity of 0.2 m s$^{-1}$ corresponds to a cycle-averaged volumetric flow rate of 5.89 L min$^{-1}$ for the equivalent case of a three-dimensional (3D) geometry with a circular cross-section. The pressure at the four outlets is modeled by a three-element Windkessel model (Westerhof et al. 2008).

### Thrombus growth model

The constitutive model of Menichini and Xu (2016) is adapted to model thrombus formation in the FL. The model controls the formation based on the wall shear stress $\tau _\mathrm {w}$, the shear rate $\dot{\gamma }$, the residence time ${T}_{\mathrm {R}}$, the concentration of coagulant *c*, the concentration of resting $c_{\mathrm {RP}}$, activated $c_{\mathrm {AP}}$, and bounded platelets $c_{\mathrm {BP}}$. Since the model employs cycle-averaged field variables that decouple the growth rates from actual reaction rates of the blood constituents involved, it achieves reasonable simulation times for thrombus formation.

The spatial and temporal averages are denoted as $\overline{\square }$ and $\langle \square \rangle$, respectively. Cycle-averaged field variables are defined as

9 $$\begin{aligned} \langle {\square }\rangle = \frac{1}{T} \int _{nT}^{(n+1)T} \square (x,y,z,t) \mathrm {d}t\,, \end{aligned}$$

where *T* is the cardiac cycle period while $n\in \mathbb {N}_0$ represents the cycle counter. Similarly, the spatial average considers normalized integrals over the domain volume *V*. By exception, the cycle-averaged residence time is additionally normalized by its maximum value in the corresponding cycle, i.e.

10 $$\begin{aligned} \langle {{T}_{\mathrm {R}}}\rangle = \frac{1}{\mathrm {max}\,({T}_{\mathrm {R}})} \frac{1}{T} \int _{nT}^{(n+1)T} {T}_{\mathrm {R}}(x,y,z,t) \mathrm {d}t\,. \end{aligned}$$

The amount of thrombus formation is influenced by the threshold values of the cycle-averaged wall shear stress $\langle {\tau }_\mathrm {w}\rangle _\mathrm {t}$, shear rate $\langle \dot{\gamma }\rangle _\mathrm {t}$, residence time $\langle {T}_{\mathrm {R}}\rangle _\mathrm {t}$, and the concentrations of bounded platelets $c_\mathrm {BPt}$ and coagulant $c_\mathrm {t}$. Regions with a high concentration of bounded platelets are associated with thrombus formation, which inhibits the blood flow. This effect is captured by the modification of the momentum balance in Eq. (1). The conversion of platelets from activated to bounded is given by

11 $$\begin{aligned} \frac{\partial c_{\mathrm {BP}}}{\partial t} = k_{\mathrm {BP}} \frac{c^2}{c^2+c_{\mathrm {t}}^2} \frac{\langle {{T}_{\mathrm {R}}}\rangle ^2}{\langle {{T}_{\mathrm {R}}}\rangle ^2+\langle {T}_{\mathrm {R}}\rangle _{\mathrm {t}}^2} \frac{\langle \dot{\gamma }\rangle _{\mathrm {t}}^2}{\langle { \dot{\gamma }}\rangle ^2 + \langle \dot{\gamma }\rangle _{\mathrm {t}}^2} c_{\mathrm {AP}}\,, \end{aligned}$$

where $k_\mathrm {BP}$ is a reaction rate constant. Zero initial concentration and zero flux boundary condition at the wall is prescribed for the bounded platelets. Activated platelets are generated by activating resting platelets through the transport equations

12 $$\begin{aligned} \frac{\partial {c_{\mathrm {RP}}}}{\partial t} + \varvec{u} \cdot \nabla c_{\mathrm {RP}} =&\nabla \cdot (D_{\mathrm {p}} \nabla c_{\mathrm {RP}}) - k_{1} c_{\mathrm {RP}} c_{\mathrm {AP}} \\ \nonumber&- k_{2} c_{\mathrm {RP}} \langle {{T}_{\mathrm {R}}}\rangle \,, \end{aligned}$$

and

13 $$\begin{aligned} \frac{\partial {c_{\mathrm {AP}}}}{\partial t} + \varvec{u} \cdot \nabla c_{\mathrm {AP}} =&\nabla \cdot (D_{\mathrm {p}} \nabla c_{\mathrm {AP}}) + k_{1} c_{\mathrm {RP}} c_{\mathrm {AP}} \\ \nonumber&+ k_{2} c_{\mathrm {RP}} \langle {{T}_{\mathrm {R}}}\rangle \,, \end{aligned}$$

where $k_{1}$ and $k_{2}$ are reaction constants, while $D_{\mathrm {p}}$ denotes the diffusion coefficient of platelets. Because red blood cells have a shear-dependent effect on the transportation of platelets (Menichini and Xu 2016; Jafarinia et al. 2020), the diffusion coefficient of platelets $D_{\mathrm {p}}$ includes, besides the Brownian diffusivity $D_\mathrm {b}$, the shear rate $\dot{\gamma }$ scaled by a parameter $\beta$,

14 $$\begin{aligned} D_{\mathrm {p}} = D_{\mathrm {b}} + \beta \dot{\gamma }\,. \end{aligned}$$

The initial concentration of resting platelets is taken as 2.5 $\times$ 10$^{14}$ platelets/m$^{3}$ and the initial concentration of activated platelets is assumed to be 5 % of the resting platelets $c_\mathrm {RP}$ (Menichini and Xu 2016). For the inlet boundary condition, $c_\mathrm {RP}$ and $c_\mathrm {AP}$ have constant values equal to their initial values. On all the other boundaries, zero flux boundary conditions are considered for $c_\mathrm {RP}$ and $c_\mathrm {AP}$.

The coagulant concentration *c* represents the lumped effect of all underlying biochemical reactions in the coagulation cascade (Menichini and Xu 2016). The transport equation of the coagulant concentration is given by

15 $$\begin{aligned} \frac{\partial {c}}{\partial t} = \nabla \cdot (D_{\mathrm {c}_{\mathrm {eff}}} \nabla c) + k_{\mathrm {c}} \frac{c_{\mathrm {BP}}^2}{c_{\mathrm {BP}}^2+c_{\mathrm {BPt}}^2} \frac{\langle \dot{\gamma }\rangle _{\mathrm {t}}^2}{\langle { \dot{\gamma }}\rangle ^2 + \langle \dot{\gamma }\rangle _{\mathrm {t}}^2}\,, \end{aligned}$$

where $k_{\mathrm {c}}$ is a reaction constant. The effective diffusion coefficient $D_{\mathrm {c}_{\mathrm {eff}}}$ is diminished in regions of high cycle-averaged shear rates (Menichini and Xu 2016), i.e.

16 $$\begin{aligned} D_{\mathrm {c}_{\mathrm {eff}}}=D_{\mathrm {c}} \frac{\langle \dot{\gamma }\rangle _{\mathrm {t}}^2}{\langle { \dot{\gamma }}\rangle ^2 + \langle \dot{\gamma }\rangle _{\mathrm {t}}^2}\,, \end{aligned}$$

where $D_{\mathrm {c}}$ is the coagulant diffusivity in resting blood. The reactive source term in Eq. (15) shows that the coagulant *c* is produced when $c_\mathrm {BP}$ is sufficiently higher than its threshold value $c_{\mathrm {BPt}}$ and, in addition, when $\langle \dot{\gamma }\rangle$ is lower than its threshold value $\langle \dot{\gamma }\rangle _\mathrm {t}$. However, the decisive production of coagulant concentration, initially assumed to be zero, occurs at the vessel wall. The coagulant flux into the domain through the wall is given by

17 $$\begin{aligned} D_{\mathrm {c}_{\mathrm {eff}}} \frac{\partial c}{\partial \varvec{\mathrm {n}}}=k_{\mathrm {cw}}\left( \langle {\tau }_\mathrm {w} \rangle , c_{\mathrm {BP}}\right) \,, \end{aligned}$$

where $\varvec{\mathrm {n}}$ is the normal vector of the wall and $k_\mathrm {cw}$ is a ‘reaction rate’ which depends on the cycle-averaged wall shear stress and the concentration of bounded platelets at the wall. If the cycle-averaged wall shear stress is below its threshold value $\langle {\tau }_{\mathrm {w}} \rangle \le {0.2}$ Pa, and, simultaneously, the concentration of bounded platelets at the wall is lower than its threshold value $c_{\mathrm {BP}}\le$ 2 $\times$ 10$^{5}$ nmol/m$^{3}$, then the reaction rate $k_\mathrm {cw}$ is 2 $\times$ 10$^{4}$ nmol/m$^{2}$/s; otherwise $k_\mathrm {cw}=0$. The boundary conditions at the inlet and outlets are set to zero flux for the coagulant. Finally, the residence time ${T}_{\mathrm {R}}$ of the liquid components or the platelets in the field is determined by the following transport equation

18 $$\begin{aligned} \frac{\partial {T}_{\mathrm {R}}}{\partial t}+\varvec{u} \cdot \nabla {T}_{\mathrm {R}}=D_{{T}_{\mathrm {R}}} \nabla ^{2} {T}_{\mathrm {R}}+1\,, \end{aligned}$$

where $D_{{T}_{\mathrm {R}}}$ is the self-diffusivity of blood. The residence time is initially zero and is kept zero at the inlet boundary. On all the other boundaries, zero flux boundary conditions are considered. The values of the constants applied in the thrombus formation model are listed in Table 4. Note that, while all parameters where adopted from Menichini and Xu (2016) or Menichini et al. (2016), a different value of the bounded platelet reaction rate $k_\mathrm {BP}$ is employed. The reason is that, in the named studies, the resting and activated platelets concentrations were normalized against their initial values to avoid numerical ill-conditioning. However, no numerical ill-conditioning was observed in the current work. Therefore, the platelet concentrations are not normalized, and a different value of $k_\mathrm {BP}$ is needed to be consistent with the works of Menichini et al.

**Table 5:** Dimension of the representative morphology for the grid independency study

Absolute parameter	Value (mm)
$\mathrm {L}_\mathrm {1}$	18.2
$\mathrm {L}_\mathrm {2}$	145.7
$\mathrm {L}_\mathrm {3}$	9.1
$\mathrm {D}_\mathrm {FL}$	18.2
$\mathrm {H}_\mathrm {T1}$	11.3
$\mathrm {H}_\mathrm {T2}$	11.3
$\mathrm {D}_\mathrm {TL}$	14.3
$\mathrm {x}_\mathrm {T1}$	17.18

**Table 6:** Results of the grid independence study comparing six different grids

No.	Number of elements	$\overline{\phi }_\mathrm {th,D}$ (%)
1	$7\,695$	13.12
2	$10\,658$	11.08
3	$14\,324$	7.41
4	$20\,260$	6.01
5	$28\,038$	4.05
6	$38\,526$	4.06

An in-house python code was developed to generate the idealized geometries of TBAD as input to the blockMesh utility in the open-source software *OpenFOAM* (Weller et al. 1998). The latter operates in a 3D Cartesian coordinate system such that formally all input geometries are required in 3D with a unit depth. However, in order to solve equations in a 2D setting, it is possible to assign the boundary condition ‘empty’ to all faces normal to the third dimension (Weller et al. 1998). Therefore, idealized geometries in the current study are 3D, but with unit depth. The governing equations of the blood flow and thrombus formation model were solved with the finite volume method. Depending on the geometry, about $60\,000$ hexahedral cells were needed to achieve a suitable discretization with a structured mesh. To resolve high gradients on the wall surface, we refined the mesh towards the wall using the ‘simpleGrading’ option. A grid independence study for a representative geometry, which is similar to Fig. 1, was performed by analyzing the volume percentage of thrombus $\overline{\phi }_\mathrm {th}$ in the FL. The mesh of each geometry was adequately scaled to ensure mesh-independent solutions for all morphologies. Table 5 shows the dimensions of the representative morphology used for the grid independence study. The morphology corresponds to a case with a large entry tear proximal to the aortic arch, leading to a high momentum of the flow into the FL. Accordingly, a high velocities and high wall gradients are to be expected, which makes this morphology especially sensitive to mesh resolution. Five different meshes were regarded, where the number of elements was each time increased by about 40 %. The number of elements and the corresponding values for volume percentage of thrombus $\overline{\phi }_\mathrm {th,D}$ are listed in Table 6. The results from the refinement steps of mesh number 5 to number 6 show a marginal relative change in the output. Consequently, grid number 5 provides a sufficient refinement factor to the element size ratio for creating the meshes. Grid number 5 was subsequently transferred by adequate scaling to the other morphologies, assuming that this ensures mesh-independent results in all cases A second-order implicit scheme was used for the time discretization with a fixed time step of $\varDelta t={0.2}$ ms. The gradient discretization scheme is a second-order central difference, while a Gaussian second-order scheme was used for the divergence schemes (Weller et al. 1998). The cardiac cycle is considered to have a period of $T=1$ s. Seven cardiac cycles were simulated to obtain a time periodic flow field. The simulations were aborted after 50 cycles since the thrombus growth had ceased at that time in most simulations. Naturally, the thrombus grows until its surface reaches the zones where the hemodynamic conditions prohibit further growth. The results are in good agreement with the results of Menichini and Xu (2016). Note that the time parameter in the thrombus formation model is not an actual physical time (Menichini and Xu 2016), and we make no prediction on the true timescale of thrombus growth in this work.

### Global sensitivity analysis

**Fig. 2:**
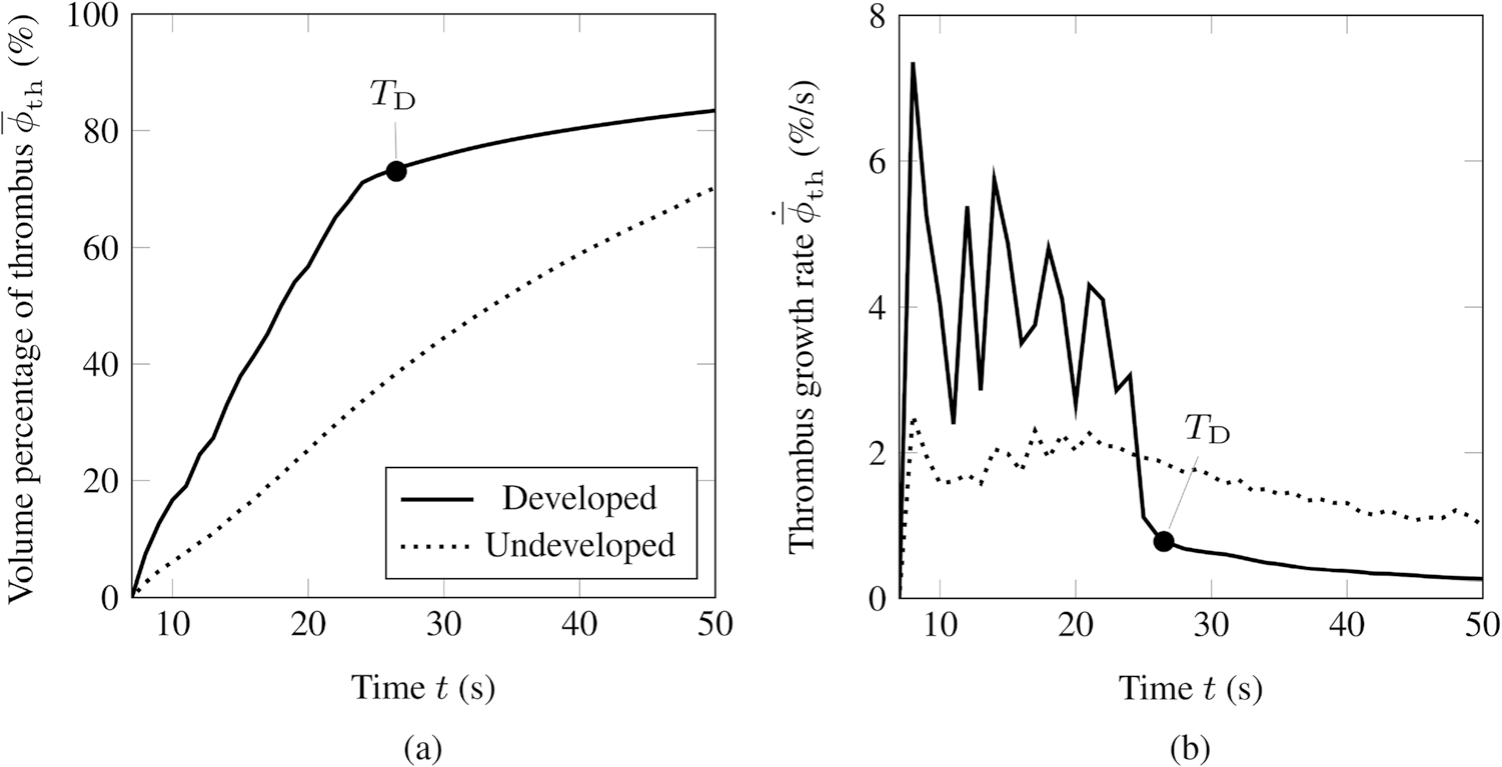
Volume percentage of thrombus $\overline{\phi }_\mathrm {th}(t)$ **a** and corresponding thrombus growth rate $\dot{\overline{\phi }}_\mathrm {th} (t)$ **b** from two random simulation results. The continuous curve indicates a developed thrombus, while an undeveloped thrombus is indicated by a dotted curve. In addition, the black circle denotes the developed time milestone $T_\mathrm {D}$

A global sensitivity analysis is employed to assess and quantify the influence of the morphological parameters of the dissected aorta on the output variables of interest, i.e. mainly to assess the degree of thrombosis (see Eq. (25), below) of the FL in the current work. Since we have no a priori knowledge about the individual or combined influence of the parameters on the output, we employ a generic variance-based approach using the so-called Sobol indices (Sobol’ 1990, 2001). For a model *f*, with a multivariate random variable, or random vector, $\varvec{x}=[x_1,\ldots ,x_i,\ldots ,x_M]$ $\in \mathbb {R}^M$ and scalar output $\mathrm {Y}=f(\varvec{x})$ $\in \mathbb {R}$, the first-order and the total-order Sobol indices, are defined, respectively, as

19 $$\begin{aligned} S_{i}&= \frac{\mathbb {V}[\mathbb {E}[\mathrm {Y} | x_i]]}{\mathbb {V}[\mathrm {Y}]}\,, \end{aligned}$$

and

20 $$\begin{aligned} S_{i}^\mathrm {T}&= { 1-\frac{\mathbb {V}[\mathbb {E}[\mathrm {Y}|\varvec{x}_{\sim i}]]}{\mathbb {V}[\mathrm {Y}]} = \frac{\mathbb {E}[\mathbb {V}[\mathrm {Y} | \varvec{x}_{\sim i}]]}{\mathbb {V}[\mathrm {Y}]} }\,, \end{aligned}$$

where $\mathbb {V}[\square ]$ and $\mathbb {E}[\square ]$ are the statistical variance and expectation operators while $[\square |\square ]$ is the conditional operator, which expresses the expectation or variance of one random variable when a second variable is given (Saltelli et al. 2008). In the conditional variance in Eq. (20) all input factors except the *i*-th are considered given, which is indicated by the tilde symbol before the index *i*. The total-order Sobol index quantifies the interactions between the factors in the computational model. The indices above describe the sensitivity of the model to its factors (Saltelli et al. 2008).

The first-order index (Eq. 19) identifies the impact of single factors on the model output. It allows defining a rank of importance among all input variables based on the influence exerted over the output. The total-order Sobol index (Eq. 20) includes the interaction between the variables of the model. Low total-Sobol indices identify the factors that do not play a decisive role in the model computation. Therefore, the total-order Sobol index is employed to select the variables that could be considered as model constants. In addition, the difference between Eqs. (19) and (20) quantifies the level of interaction between the input parameters.

The computation of the indices can be achieved with different techniques, such as the Monte Carlo sampling or the quasi-Monte Carlo sequences (Saltelli 2002; Saltelli et al. 2008). However, both require a high number of model computations to reach convergence (Iooss and Lemaître 2015). In this study, the use of a surrogate model, in particular, the polynomial chaos expansion (PCE), is employed to assess the indices from the expansion coefficients (Wiener 1938; Ghanem and Spanos 1991; Xiu and Karniadakis 2002; Sudret 2008; Le Gratiet et al. 2017).

For independent input random variables $x_i$, the vector $\varvec{x}$ can be represented by the standard random vector $\varvec{\xi }$ through an isoprobabilistic transform (Sudret 2008)

21 $$\begin{aligned} \begin{array}{ll} \varvec{x} = T(\varvec{\xi }) \,,&\quad \varvec{\xi } = \{\xi _1,\ldots , \xi _M\} \,. \end{array} \end{aligned}$$

The PCE $\widetilde{f}(\varvec{x})$ is then formulated, for a scalar output $\mathrm {Y}$ with finite variance (Xiu 2010), i.e. $\mathbb {V}[\mathrm {Y}]<\infty$, as

22 $$\begin{aligned} \widetilde{f}(\varvec{x})= \sum _{\varvec{\alpha }\in \mathcal {A}} c_{\varvec{\alpha }} \varPsi _{\varvec{\alpha }}(\varvec{\xi }) \,, \end{aligned}$$

where $\varvec{\alpha }=(\alpha _1,\ldots ,\alpha _M)$ is a multi-index defined in $\mathcal {A} \subseteq \mathbb {Z}_0^M$, $c_{\varvec{\alpha }}$ are polynomial coefficients, and $\varPsi _{\varvec{\alpha }}(\varvec{\xi })$ are multivariate polynomials. The multivariate polynomials are computed, for the standard random vector $\varvec{\xi }$, as a product of univariate polynomials $\psi _{\alpha _i}$ as

23 $$\begin{aligned} \varPsi _{\varvec{\alpha }}(\varvec{\xi })=\prod _{i=1}^M \psi _{\alpha _i}(\xi _i) \,, \end{aligned}$$

where, for a polynomial of degree *p*, $\alpha _i\ge 0$, and $\sum _{i=1}^M \alpha _i\le p$, and the univariate polynomials $\psi _{\alpha _i}(\xi _i)$ are orthogonal to the *i*-th input probability distribution function (Wiener 1938; Ghanem and Spanos 1991; Xiu and Karniadakis 2005; Xiu 2010).

In the variance-based method, the PCE terms identify the variance decomposition of the model. The formulation is usually truncated such that the PCE sufficiently explains the model variance $\sigma ^2_\mathrm {Y}$, which is given by

24 $$\begin{aligned} \sigma ^2_\mathrm {Y} = \sum _{\varvec{\alpha }\in \mathcal {A}} c_{\varvec{\alpha }}^2\,. \end{aligned}$$

**Fig. 3:**
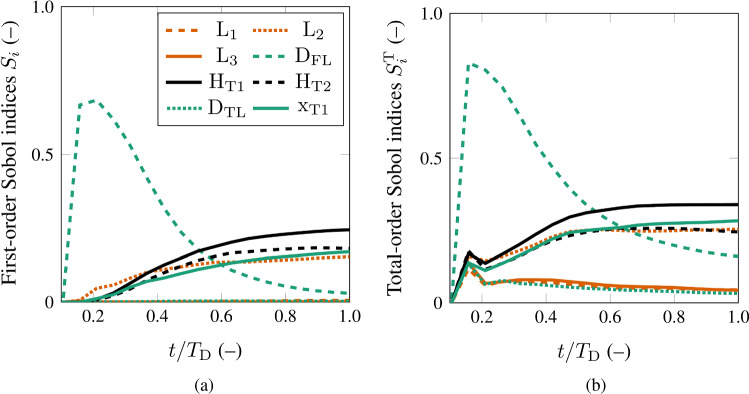
Results of the sensitivity analysis for the volume percentage of the thrombus $\overline{\phi }_\mathrm {th}(t)$ in the FL. The results represent the first-order Sobol indices **a** and the total-order Sobol indices **b** for all the absolute morphological parameters

For the sensitivity analysis, several morphologies of a dissected aorta are analyzed. The sensitivity analysis is performed on the absolute morphological parameters. In particular, the considered input factors are the lengths of the FL area, which is subdivided into $\mathrm {L}_\mathrm {1}$, $\mathrm {L}_\mathrm {2}$, and $\mathrm {L}_\mathrm {3}$, the TL and FL diameters, $\mathrm {D}_\mathrm {TL}$ and $\mathrm {D}_\mathrm {FL}$, the size of the proximal and distal tears, $\mathrm {H}_\mathrm {T1}$ and $\mathrm {H}_\mathrm {T2}$, and the proximal tear location $\mathrm {x}_\mathrm {T1}$. The factors are taken to follow a uniform probability distribution, the ranges of which are given in Table 2.

To sufficiently represent the input variability, the input sample space of the global sensitivity analysis has the dimension $N_s = 4\,000$ and is sampled with a Latin hypercube sampling method (Tang 1993). For this study, Legendre polynomials are employed for the PCE formulation due to the uniform distribution of the input parameters (Xiu 2010). The simulations were performed with HPC resources provided the ZID of Graz University of Technology, where each simulation took about four hours using eight cores. The PCE is calculated with the *Matlab* toolbox *UQlab* (Marelli and Sudret 2014). The generalized Sobol indices are implemented for the time-dependent output (Alexanderian et al. 2020). The indices are computed from the surrogate model, solved with a last-angle regression and the hyperbolic index of $q=0.95$ (Blatman and Sudret 2010, 2011). The polynomial degree is chosen to have the lowest leave-one-out error $\varepsilon _\mathrm {LOO}$ (Blatman and Sudret 2010; Le Gratiet et al. 2017) for a range of degrees $3 \le p \le 8=M$.

### Quantities of interest

The volume percentage of the thrombus $\overline{\phi }_\mathrm {th}(t)$ and the growth rate $\dot{\overline{\phi }}_\mathrm {th} (t)$ have concise information about the FL thrombosis status to account for thrombus formation in the FL (Jafarinia et al. 2020; Melito et al. 2020). The volume percentage of thrombus $\overline{\phi }_\mathrm {th}(t)$ is defined by Jafarinia et al. (2020)

25 $$\begin{aligned} \overline{\phi }_\mathrm {th}(t) = \frac{100}{V_\mathrm {FL}} \int _{V_\mathrm {FL}} \phi _\mathrm {th}(x,y,z,t) \mathrm {d}V_\mathrm {FL}\,, \end{aligned}$$

where $V_\mathrm {FL}$ is the volume of the FL. However, in this study, due to the chosen 2D idealized geometries, the volume $V_\mathrm {FL}$ is expressed as FL area $A_\mathrm {FL}$ multiplied with the unit depth. The time derivative of Eq. (25) gives the thrombus growth rate $\dot{\overline{\phi }}_\mathrm {th} (t)$.

Figure 2(a) shows the volume percentage of thrombus and Fig. 2(b) the corresponding growth rate for two representative simulations. The thrombus in the FL is defined as ‘developed’ when the growth rate meets a predefined set of criteria. A developed thrombus shows a particular and well-defined trend in $\overline{\phi }_\mathrm {th}(t)$. Its curve can be divided into two main parts: an initial steep and linear growth, which is then followed by another linear, but much slower, thrombus growth. This behavior is visible in Fig. 2(a) by the solid curve while the thrombus growth did not reach a developed state in the case corresponding to the dotted curve. For most simulations, the volume percentage of thrombus reaches a developed state at some point in the simulation and does not show any successive substantial change. After defining a developed thrombus, we removed simulation results where $\overline{\phi }_\mathrm {th}(t)$ did not represent the defined developed state at the end of the simulation. As indicated in Fig. 2(a) and (b), the point in the simulation at which a thrombus reaches a developed state is denoted as $T_\mathrm {D}$.

**Fig. 4:**
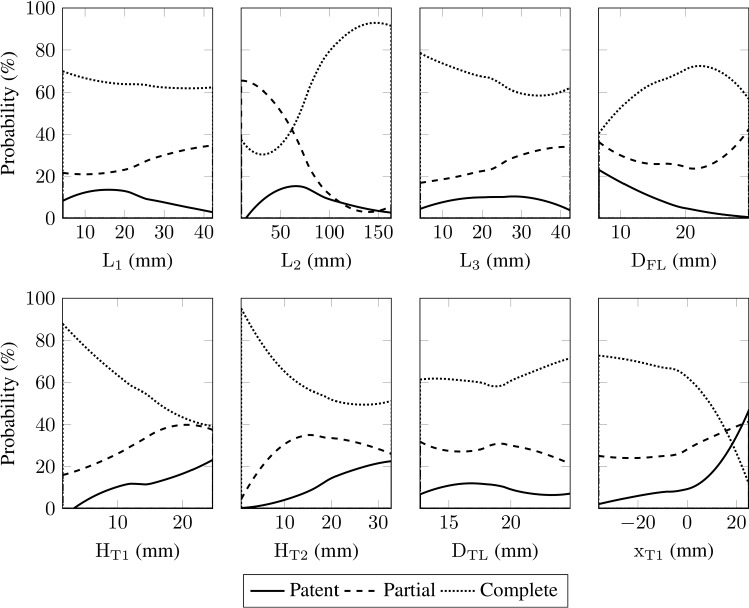
Probability of developing a patent, a partially thrombosed, or a completely thrombosed FL for each absolute morphological parameter

The time $T_\mathrm {D}$ is determined by three criteria. To begin with, we state that a thrombus shall only be considered developed if the thrombus growth rate is characterized by a peak followed by a reduction of growth rate, eventually reaching a plateau of a much smaller growth rate than at the peak. To identify developed states and to stably and automatically determine the time $T_\mathrm {D}$, we combine an absolute criterion on the growth rate with a relative criterion based on the drop relative to the peak value with the requirement that there is no strong relative variation in the growth rate subsequently. The following three criteria are used: (i) the first criterion requires that the thrombus growth rate needs to be below 5 % s$^{-1}$. If the growth rate is smaller than 5 % s$^{-1}$, the time $T_\mathrm {D}$ is identified as the smallest time at which (ii) the growth rate fell below 30 % of the maximal growth rate observed in the simulation, and (iii) the relative deviation of the growth rate during the following two seconds from its value at $T_\mathrm {D}$ is at most 0.75. If these three criteria are never met simultaneously in a simulation, the thrombus is not considered as having reached a developed state. Unless otherwise specified, the term ‘formed thrombus’ always refers to the developed thrombus, i.e. the volume percentage of developed thrombus $\overline{\phi }_\mathrm {th,D}$ at time $T_\mathrm {D}$.

## Results

### Sensitivity analysis on thrombus formation

Figure 3(a) and (b) show the generalized Sobol indices for the volume percentage of thrombus $\overline{\phi }_\mathrm {th}(t)$ versus the normalization of the time $t/T_\mathrm {D}$. The simulation time was normalized with the developed time milestone to ensure that the sensitivity analysis is performed on the volume percentage of developed thrombus $\overline{\phi }_\mathrm {th,D}$ until the developed time milestone $T_\mathrm {D}$, see Fig. 2(a) and (b).

**Fig. 5:**
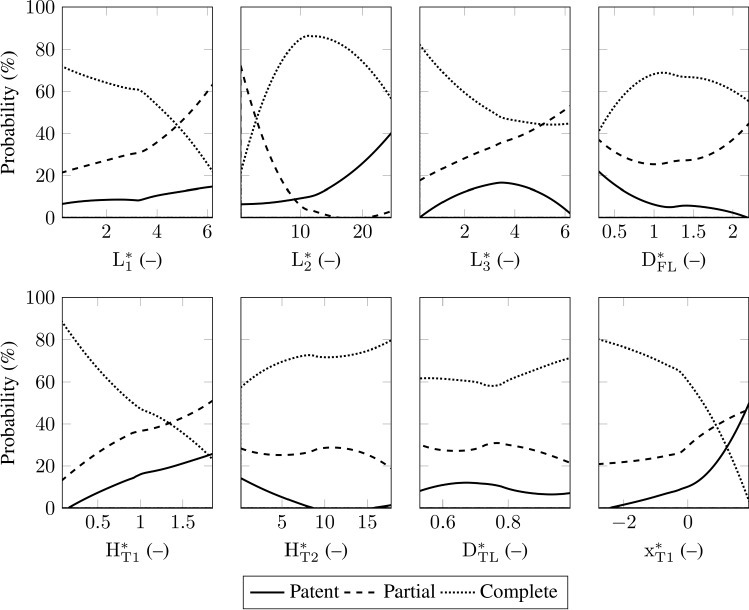
Probability of developing a patent, a partially thrombosed or a completely thrombosed FL for each dimensionless morphological parameter

The volume percentage of thrombus $\overline{\phi }_\mathrm {th}(t)$ is significantly affected by the FL diameter $\mathrm {D}_\mathrm {FL}$. The FL diameter has the most significant role at the initial stage of thrombus formation, which can be seen by the first-order Sobol indices in Fig. 3(a). However, as the thrombus formation progresses in time, the impact of the FL diameter decreases. At about half of the normalized time, the influence of the tears sizes $\mathrm {H}_\mathrm {T1}$ and $\mathrm {H}_\mathrm {T2}$ becomes dominant. Immediately thereafter, the influence of the proximal tear location $\mathrm {x}_\mathrm {T1}$ and the medial FL length $\mathrm {L}_\mathrm {2}$ exceeds the impact of FL diameter, although the influence of the former parameters is still less significant than that of the size of the tears. This change of influence on $\overline{\phi }_\mathrm {th}(t)$ divides the thrombus formation into two phases: an initial and a consecutive thrombus development phase. The initial phase lasts from thrombus initiation till about fifty percent of the developed time $T_\mathrm {D}$, whereas the consecutive thrombus development phase includes the remaining process until $t/T_\mathrm {D}=1$.

The total-order Sobol indices in Fig. 3(b) suggest that the volume percentage of thrombus $\overline{\phi }_\mathrm {th}(t)$ is not influenced by the FL segment lengths $\mathrm {L}_\mathrm {1}$ and $\mathrm {L}_\mathrm {3}$, and the TL diameter $\mathrm {D}_\mathrm {TL}$. The interaction between all the morphological parameters of the model is evaluated through the difference between the first and total Sobol indices. These values are relatively low, so we conclude that the interaction is only of minor importance.

### Morphology classification based on patent, partially and completely thrombosed false lumina

**Fig. 6:**
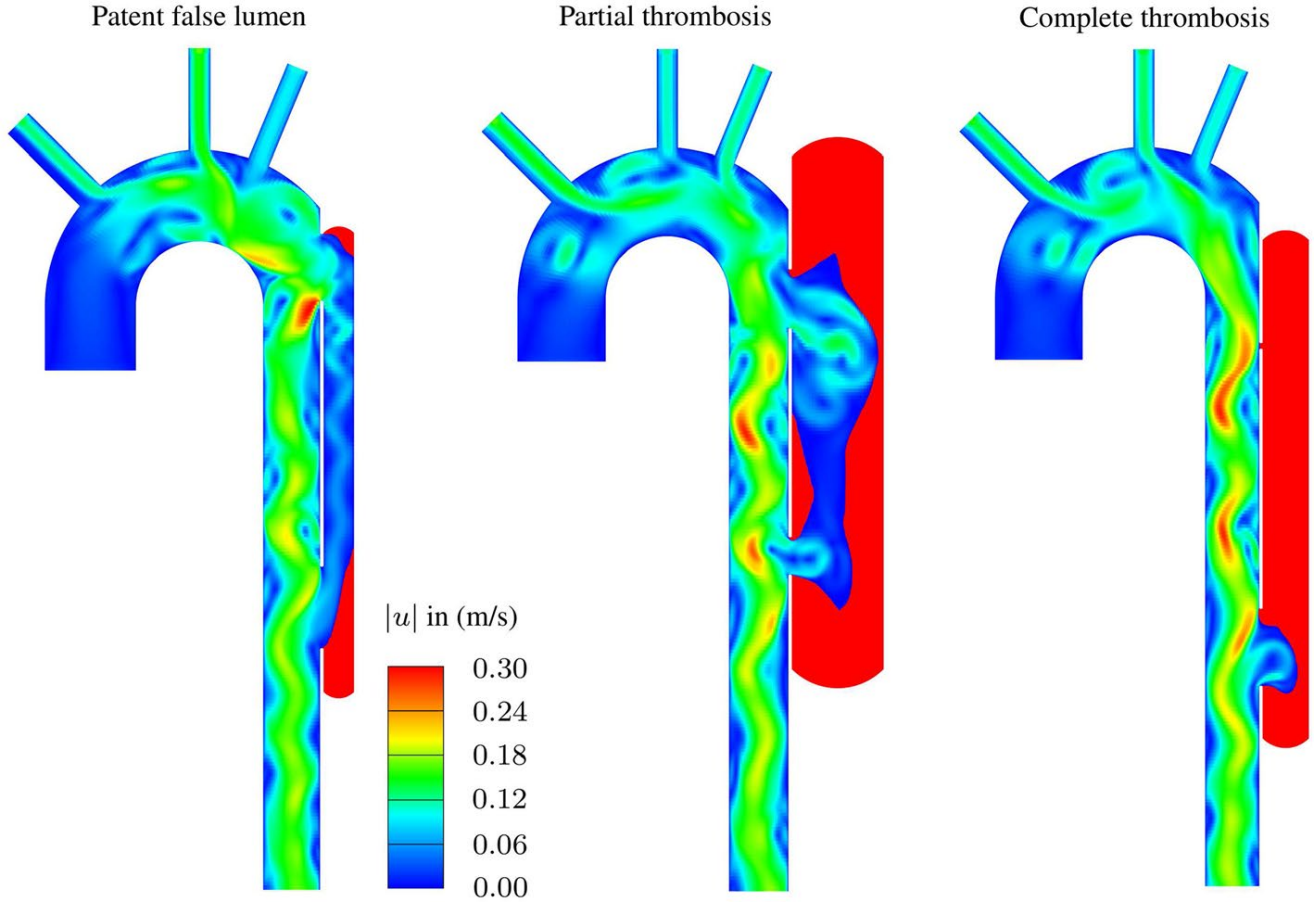
Representative results from CFD analyses showing color contours of velocity magnitude and indicating thrombus formation in the FL: Patent FL, partially thrombosed FL, and completely thrombosed FL. The red zones in the FL represent the predicted thrombus

Following the literature, the definition of the FL status is based on the flow and the presence of thrombus in the FL (Tsai et al. 2007; Trimarchi et al. 2013). In this study, we introduce the definition of the FL status based on the amount of thrombus in the FL indicated by the volume percentage of developed thrombus $\overline{\phi }_\mathrm {th,D}$. The probability distribution of the output $\overline{\phi }_\mathrm {th,D}$ was investigated to quantify the status of FL thrombosis. The investigation showed that the morphology variations separate the probability distribution into three distinct regions. This suggested that three sets of morphologies lead to distinct probability distributions. Hence, we classified these three regions as patent, partially thrombosed, and completely thrombosed FL. Other classification criteria were investigated for the quantification of FL status. However, the produced results were hardly interpretable. In the chosen classification, for $\overline{\phi }_\mathrm {th,D}< 30$ %, the FL status is defined as patent, i.e. the blood flow is fully guaranteed due to the lack of a thrombus. The second class is defined for 30 % $\le \overline{\phi }_\mathrm {th,D}\le 70$ %, where the FL is considered partially thrombosed. In other words, the blood can still partially flow in the FL. And finally, for $\overline{\phi }_\mathrm {th,D}> 70$ %, we describe the FL status as completely thrombosed. Here, the blood flow rate in the FL approaches zero. Patent, partially thrombosed, and completely thrombosed FL account for about 10 %, 30 %, and 60 % of the simulations performed, respectively. Based on these definitions, the influence of the morphological parameters on the probability of having the respective FL status is examined. Therefore, for each FL status, we focus on the morphological parameters to understand which parameters favor a complete thrombosis and, in addition, which parameters increase the risk of having a patent and a partially thrombosed FL.

In Figs. 4 and 5, the fractions (in percentage) of the simulations resulting in a patent, partially thrombosed or completely thrombosed FL are displayed as functions of the morphological parameters. The results demonstrate that the medial FL length $\mathrm {L}_\mathrm {2}$, the FL diameter $\mathrm {D}_\mathrm {FL}$, the proximal tear size $\mathrm {H}_\mathrm {T1}$, the distal tear size $\mathrm {H}_\mathrm {T2}$, and the proximal tear location $\mathrm {x}_\mathrm {T1}$ have strong influence on the FL status. This finding is in good agreement with the sensitivity analysis results presented before (Sect. 3.1). As shown in Fig. 3, these five parameters were the most sensitive parameters and are primarily responsible for the output variation. Other morphological parameters, i.e. the proximal and distal FL length, $\mathrm {L}_\mathrm {1}$ and $\mathrm {L}_\mathrm {3}$, and the TL diameter $\mathrm {D}_\mathrm {TL}$, do not influence the probability of having a respective FL status. This is also consistent with the low total-order Sobol indices reported in Sect. 3.1.

#### Morphologies favoring patent false lumina

The probability of having a patent FL noticeably increases with the size of the tears $\mathrm {H}_\mathrm {T1}$ and $\mathrm {H}_\mathrm {T2}$, and the proximal tear location $\mathrm {x}_\mathrm {T1}$. But an increase in FL diameter $\mathrm {D}_\mathrm {FL}$ reduces the risk of having a patent FL to nearly 0 %. The FL length $\mathrm {L}_\mathrm {2}$ shows a non-monotonic influence on the patency, since for long and short distances between the tears, the probability of a patent FL slightly decreases. Regarding the dimensionless parameters, see Fig. 5, the FL patency is affected by the medial slenderness $\mathrm {L}_\mathrm {2}^*$, the proximal tear aspect ratio $\mathrm {H}_\mathrm {T1}^*$, and the proximal tear deviation $\mathrm {x}_\mathrm {T1}^*$. An increase in these parameters increases the probability of having a patent FL from 0 % up to 50 %. Interestingly, the probability of a patent FL increases at $\mathrm {x}_\mathrm {T1}^*=0$ and $\mathrm {L}_\mathrm {2}^*=15$, where $\mathrm {x}_\mathrm {T1}^*=0$ corresponds to the proximal tear location at the level of the center of the aortic arch, see Fig. 1. The FL-to-TL diameter ratio $\mathrm {D}_\mathrm {FL}^*$ and the distal to proximal tear size ratio $\mathrm {H}_\mathrm {T2}^*$ cause a considerable reverse influence. For the case that $\mathrm {H}_\mathrm {T2}^*\ge 8$, the probability of having a patent FL is about zero. Also, for the case of $\mathrm {D}_\mathrm {FL}^*\ge 2$, no patent FL was recorded, whereas for low values, i.e. about $\mathrm {D}_\mathrm {FL}^*=0.5$, the probability is 20 %. The distal slenderness indicates a maximum probability at $\mathrm {L}_\mathrm {3}^*=3.5$. When moving away from this maximum, this value reduces the risk of a patent FL. The proximal slenderness $\mathrm {L}_\mathrm {1}^*$ and the TL to arch diameter ratio $\mathrm {D}_\mathrm {TL}^*$ show only slight variations in the probability.

**Fig. 7:**
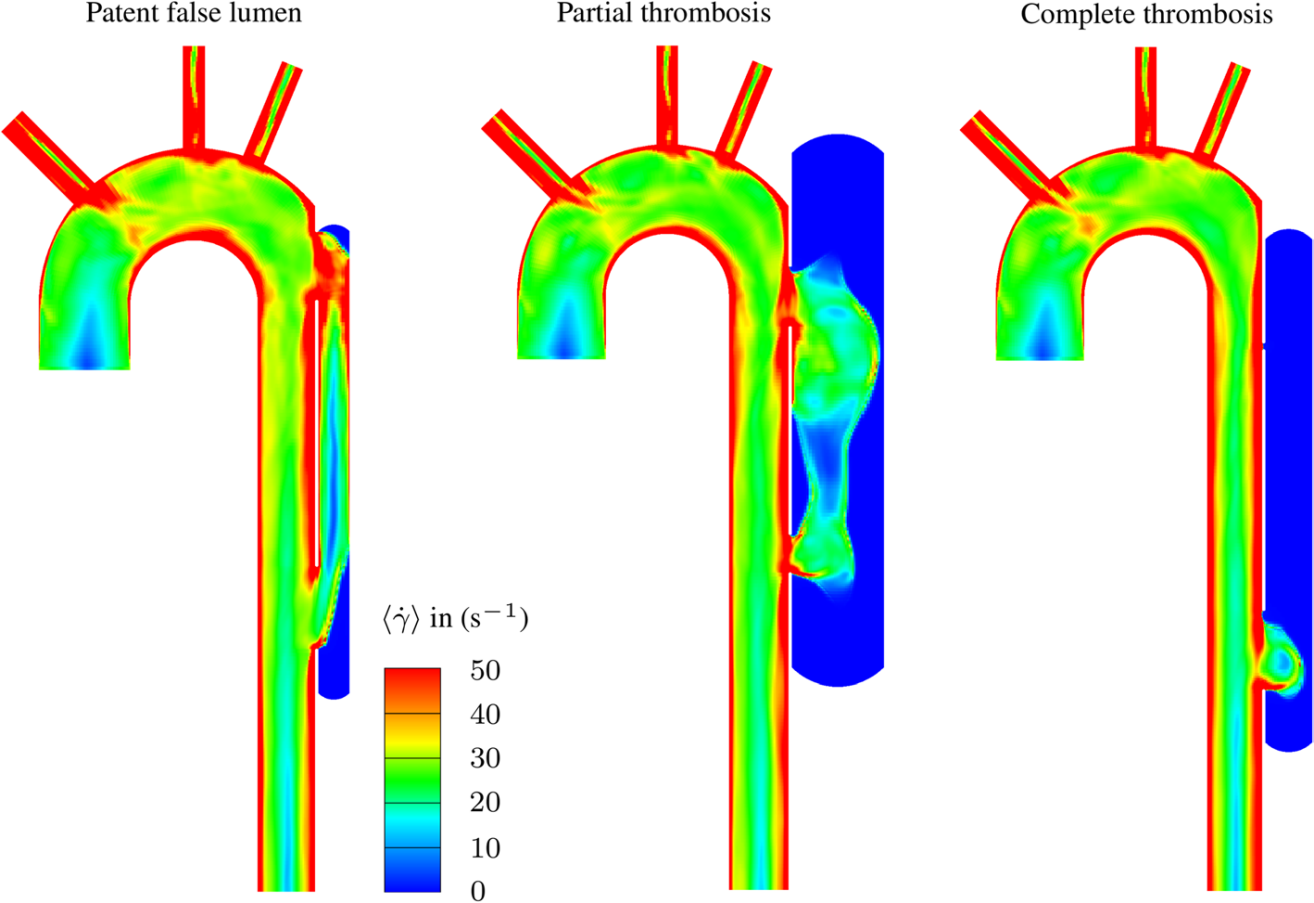
Representative results from CFD analyses showing cycle-averaged shear rate $\langle \dot{\gamma }\rangle$ color contours: Patent FL, partially thrombosed FL, and completely thrombosed FL

In summary, a patent FL is more likely for morphologies with higher values of medial slenderness $\mathrm {L}_\mathrm {2}^*$, proximal tear aspect ratio $\mathrm {H}_\mathrm {T1}^*$, proximal tear deviation $\mathrm {x}_\mathrm {T1}^*$ and lower values of FL-to-TL diameter ratio $\mathrm {D}_\mathrm {FL}^*$ and the distal to proximal tear size ratio $\mathrm {H}_\mathrm {T2}^*$.

#### Morphologies favoring partially thrombosed false lumina

The probability of having a partial thrombosis in the FL is raised by increased values of the tear sizes $\mathrm {H}_\mathrm {T1}$ and $\mathrm {H}_\mathrm {T2}$. Large FL diameters $\mathrm {D}_\mathrm {FL}$ also contribute to an increased risk of a partially thrombosed FL. In addition, the medial distance between the tears $\mathrm {L}_\mathrm {2}$ reduces the risk of partial thrombosis significantly. In contrast, the proximal tear location $\mathrm {x}_\mathrm {T1}$ has low influence on the development of a partially thrombosed FL.

The probability of a partial thrombosis increases with increasing value of the distal slenderness $\mathrm {L}_\mathrm {3}^*$ and the proximal tear aspect ratio $\mathrm {H}_\mathrm {T1}^*$ from about 10 % to 50 %. In addition, it increases with the proximal slenderness $\mathrm {L}_\mathrm {1}^*$ and the FL-to-TL diameter ratio $\mathrm {D}_\mathrm {FL}^*$ if $\mathrm {L}_\mathrm {1}^*\ge 3.5$ and if the TL diameter is larger than the FL diameter, i.e. $\mathrm {D}_\mathrm {FL}^*\ge 1$. The only reverse trend occurs for values of $\mathrm {L}_\mathrm {2}^*$ between 1 and 15, where the probability of partial thrombosis drops from about 60 % to 0 %, where the probability is almost zero for $\mathrm {L}_\mathrm {2}^*>15$. A slight decrease in the risk of partial thrombosis can be seen when the distal tear is more than eight times larger than the proximal tear, $\mathrm {H}_\mathrm {T2}^*\ge 8$. An increasing proximal tear deviation $\mathrm {x}_\mathrm {T1}^*$ increases the probability of partial thrombosis by about 30 percentage points. While the TL to arch diameter ratio $\mathrm {D}_\mathrm {TL}^*$ does not influence the probability noticeably.

In conclusion, higher values of the proximal and distal slenderness $\mathrm {L}_\mathrm {1}^*$ and $\mathrm {L}_\mathrm {3}^*$, the FL-to-TL diameter ratio $\mathrm {D}_\mathrm {FL}^*$, and the proximal tear aspect ratio $\mathrm {H}_\mathrm {T1}^*$, along with lower values of medial slenderness $\mathrm {L}_\mathrm {2}^*$ imply a more likely occurrence of partial thrombosis.

**Fig. 8:**
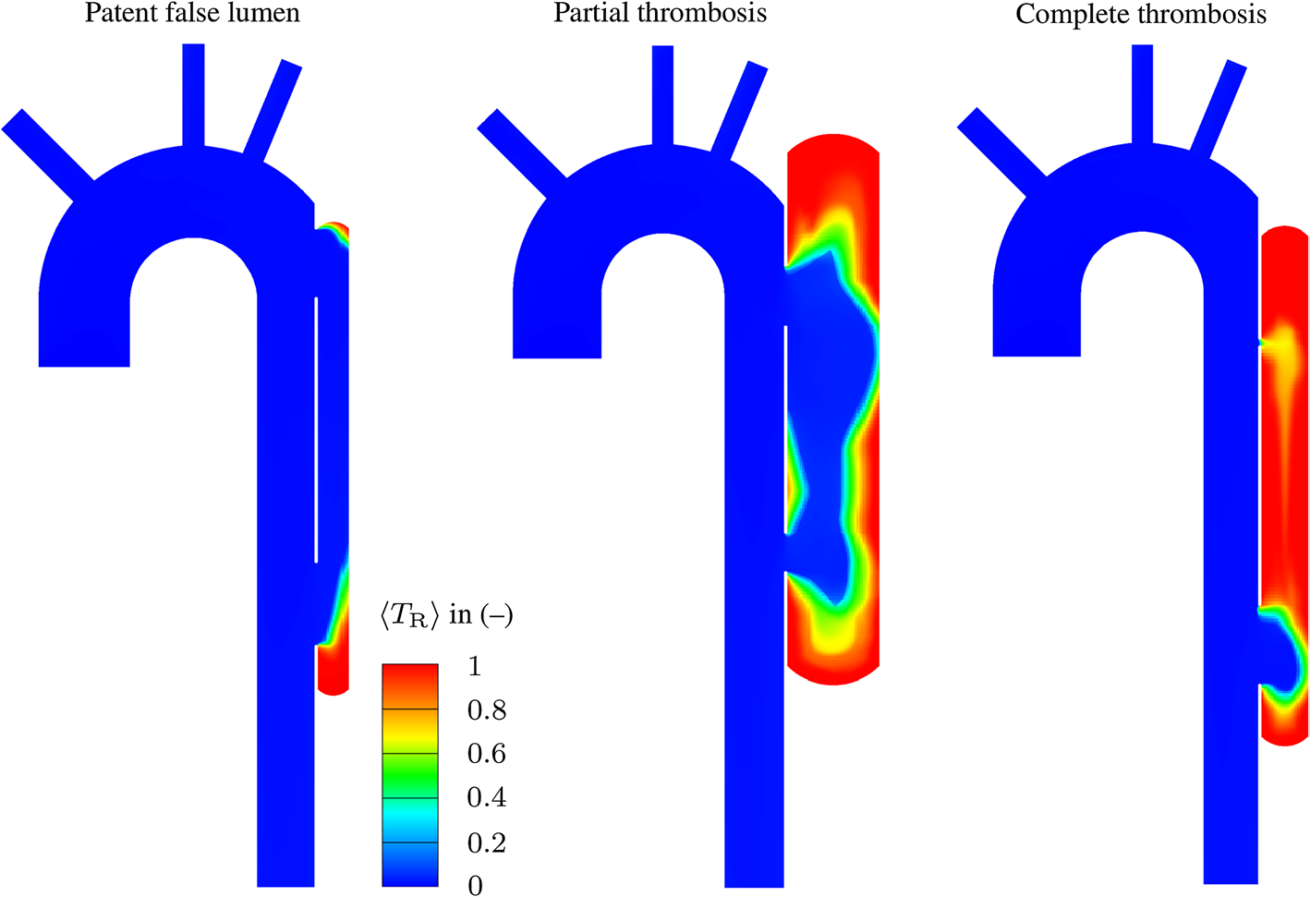
Representative results from CFD analyses showing cycle-averaged residence time $\langle {T}_{\mathrm {R}}\rangle$ color contours: Patent FL, partially thrombosed FL, and completely thrombosed FL

**Table 7:** An example of the combination of dimensionless morphological parameters that results in the three thrombosis status of the FL: Patent FL, partially thrombosed FL, and completely thrombosed FL

FL status	$\mathrm {L}_\mathrm {1}^*$	$\mathrm {L}_\mathrm {2}^*$	$\mathrm {L}_\mathrm {3}^*$	$\mathrm {D}_\mathrm {FL}^*$	$\mathrm {H}_\mathrm {T1}^*$	$\mathrm {H}_\mathrm {T2}^*$	$\mathrm {D}_\mathrm {TL}^*$	$\mathrm {x}_\mathrm {T1}^*$	$\overline{\phi }_\mathrm {th,D}$
	(–)	(–)	(–)	(–)	(–)	(–)	(–)	(–)	(%)
Patent FL	1.13	11.20 $\uparrow$	2.90 $\updownarrow$	0.53 $\downarrow$	1.20 $\uparrow$	1.22 $\downarrow$	0.62	0.66 $\uparrow$	13
Partial thrombosis	1.55 $\uparrow$	2.80 $\downarrow$	1.23 $\uparrow$	1.55 $\uparrow$	1.02 $\uparrow$	0.64	0.67	− 0.02 $\uparrow$	54
Complete thrombosis	2.30 $\downarrow$	6.53 $\updownarrow$	1.98 $\downarrow$	0.85 $\updownarrow$	0.13 $\downarrow$	11.27 $\uparrow$	0.62	− 0.91 $\downarrow$	84

The arrows indicate the direction to obtain a higher probability for the respective thrombosis status of the FL, i.e. ($\downarrow$) indicates lower values, ($\uparrow$) indicates higher values, and ($\updownarrow$) indicates non-monotonous behavior in the predefined probability range

#### Morphologies favoring completely thrombosed false lumina

A completely thrombosed FL is more likely to appear for smaller proximal and distal tear sizes $\mathrm {H}_\mathrm {T1}$ and $\mathrm {H}_\mathrm {T2}$, along with a more distal entry tear $\mathrm {x}_\mathrm {T1}$ ($\mathrm {x}_\mathrm {T1}<0$) and longer medial FL length $\mathrm {L}_\mathrm {2}$. Also, the FL diameter $\mathrm {D}_\mathrm {FL}$ does not show a monotonic behavior. Overall, up to an FL diameter of $\mathrm {D}_\mathrm {FL}=$ 22 mm, the larger the FL diameter is, the higher the probability of a complete thrombosis of the FL becomes. With a larger FL diameter, the probability then decreases slightly. The probability of a complete thrombosis increases from 60 % to 80 % for higher values of $\mathrm {H}_\mathrm {T2}^*$. A similar increase in the likelihood of a complete thrombosis is seen when the TL to arch diameter ratio is $\mathrm {D}_\mathrm {TL}^*\ge 0.7$. The probability of a complete FL thrombosis clearly decreases with an increase in the proximal tear aspect ratio $\mathrm {H}_\mathrm {T1}^*$ and the proximal tear deviation $\mathrm {x}_\mathrm {T1}^*$ from 80 % to 20 % and 0 %, respectively. In contrast, no chance of a complete thrombosis exists for values of $\mathrm {x}_\mathrm {T1}^*\approx 2$. A rapid decrease in the probability is also seen for $\mathrm {L}_\mathrm {1}^*\ge 3.5$, whereas for $\mathrm {L}_\mathrm {1}^*\le 3.5$, the probability variation is negligible. The distal slenderness $\mathrm {L}_\mathrm {3}^*$ has a reverse impact on complete thrombosis for values lower than 3.5. Here, too, the critical value of $\mathrm {D}_\mathrm {FL}^*=1$ plays a decisive role. Note that for $\mathrm {D}_\mathrm {FL}^*\le 1$ an increase in $\mathrm {D}_\mathrm {FL}^*$ elevates the probability of a complete thrombosis and for $\mathrm {D}_\mathrm {FL}^*\ge 1$ it decreases. Medial slenderness $\mathrm {L}_\mathrm {2}^*$ also shows a dramatic change in the probability for a completely thrombosed FL, reversing the trend at about $\mathrm {L}_\mathrm {2}^*=$ 10.

To conclude, our results suggest that a completely thrombosed FL is most likely when the proximal tear aspect ratio $\mathrm {H}_\mathrm {T1}^*$, proximal tear deviation $\mathrm {x}_\mathrm {T1}^*$, proximal slenderness $\mathrm {L}_\mathrm {1}^*$, and distal slenderness $\mathrm {L}_\mathrm {3}^*$ are lower values, the distal to proximal tear ratio has high values, and the medial slenderness $\mathrm {L}_\mathrm {2}^*$ is far from its extreme values.

**Table 8:** Value of PCE coefficients $c_i$ and sum of percentage of explained variance $\sigma ^2_{\mathrm {Y}k}$ of the PCE surrogate of $\overline{\phi }_\mathrm {th,D}$

	$c_0$	$c_1$	$c_2$	$c_3$	$c_4$	$c_5$	$c_6$	$c_7$	$\ldots$	$c_\infty$
(–)	57.22	$-12.06$	$-10.32$	9.62	$-9.48$	2.92	2.50	2.40	$\ldots$	$5.05 \cdot 10^{-5}$
$\sigma ^2_{\mathrm {Y}k}$ (%)	–	25.40	44.00	60.10	75.80	77.30	78.45	79.50	$\ldots$	100

### Example of classified morphologies

Based on the classification of the morphologies, we selected three combinations of dimensionless parameters to show how the morphologies affect the FL thrombosis status, as shown in Table 7. For this purpose, arrows indicate the direction to obtain a higher probability of the respective thrombosis status of the FL, i.e. ($\downarrow$) means lower values, ($\uparrow$) means higher values, and ($\updownarrow$) does not indicate monotonous behavior in the predefined probability range. The morphologies, the velocity magnitude and the thrombus formation in the FL are illustrated in Fig. 6 for each combination. In the patent FL, large proximal and distal tears and the FL slenderness create a hemodynamic condition that prevents thrombus growth. By contrast, in the partial thrombosed FL, about 60 % of the FL is filled with thrombus. Finally, a small proximal tear causes a low flow exchange between the lumina favoring complete thrombosis. After the thrombus starts forming on the FL wall due to low cycled-averaged wall shear stress, the bulk thrombus growth is mainly driven by shear rate and residence time (Menichini and Xu 2016; Menichini et al. 2016; Jafarinia et al. 2020). Figs. 7 and 8 show the color contours of cycle-averaged shear rate $\langle \dot{\gamma } \rangle$ and residence time $\langle {T}_{\mathrm {R}}\rangle$, respectively, for the three case. Low residence time and high shear rate prevent thrombus formation near and between the tears in the patent and partially thrombosed FL. In the completely thrombosed FL there is only a small zone in the vicinity of the distal tear where the residence time is low and the shear rate is high. Therefore, the thrombus does not form in this area.

The thrombus formation model, computed for various TBAD morphologies, has been analyzed through the generation of a surrogate model, namely the PCE. More information regarding the PCE formulation and construction are reported in Sect. 2.4. To improve the insight into the model, the PCE surrogate model of the volume percentage of thrombus $\overline{\phi }_\mathrm {th,D}$ is reported and truncated up to the seventh term of the 315 nonzero coefficients. The complete list of PCE coefficients and polynomials degree is reported in the supplementary material. The formulation is detailed in Eq. (26) where the volume percentage of thrombus $\overline{\phi }_\mathrm {th,D}$ is expressed as a function of the morphological parameters. With abuse of notation, the morphological parameters reported in Eq. (26) represent the standard variables $\xi _i$ of the univariate Legendre polynomials $\varPsi _{\alpha _i}(\xi _i)$. Table 8 lists the polynomial coefficients $c_i$ and the variance of the output $\sigma _\mathrm {Y}$ computed from Eq. (24). The truncation of the surrogate PCE can explain about 80 % of the total outcome variability. Moreover, in Table 8, we list the cumulative sum of the explained variance $\sigma ^2_{\mathrm {Y}k} = \sum _{i=1}^k c_i^2$ in percentage. The PCE metamodel is formulated as follows

26 $$\begin{aligned} \overline{\phi }_\mathrm {th,D}\approx&\, c_0 + c_1 \mathrm {H}_\mathrm {T1}+ c_2 \mathrm {x}_\mathrm {T1}+ c_3 \mathrm {L}_\mathrm {2}+ c_4 \mathrm {H}_\mathrm {T2}\nonumber \\&+ c_5 \left( \frac{1}{4} - \frac{3}{4} \mathrm {x}_\mathrm {T1}^2 + \mathrm {D}_\mathrm {FL}^2 \left( -\frac{3}{4} + \frac{9}{4} \mathrm {x}_\mathrm {T1}^2 \right) \right) \nonumber \\&+ c_6 \left( -\frac{1}{2} + \frac{3}{2} \mathrm {H}_\mathrm {T2}^2 \right) + \frac{c_7}{2} \mathrm {x}_\mathrm {T1}+ \frac{3}{2} c_7 \mathrm {H}_\mathrm {T2}^2 \mathrm {x}_\mathrm {T1}\,. \end{aligned}$$

## Discussion

In this study, we proposed a systematic approach to identify the most significant morphological parameters affecting FL thrombosis. To develop our approach, we started with idealized geometries. It is known that idealized geometries provide physiologically relevant information in both 2D and 3D  (Tsai et al. 2008; Ben Ahmed et al. 2016; Menichini and Xu 2016; Menichini et al. 2016; Salameh et al. 2019; Zadrazil et al. 2020; Jafarinia et al. 2020; Keramati et al. 2020). In the current work, the choice of an idealized geometry was essential to allow sufficient statistics for the global sensitivity analysis. The presented results improve the understanding of the individual and combined influence of morphological parameters on the thrombus formation process and the final status of FL thrombosis.

In this study we considered a number of parameters to describe the morphology of a TBAD. These morphological parameters were assumed to follow a uniform probability distribution covering a wide variety of morphologies. This simple assumption was made due to the lack of clinical data on the actual probability distribution of the parameters in patients diagnosed with TBAD.

As indicated by the results, the most critical morphological parameters influencing the level of thrombosis in the FL are the proximal tear size $\mathrm {H}_\mathrm {T1}$, the distal tear size $\mathrm {H}_\mathrm {T2}$, the proximal tear location $\mathrm {x}_\mathrm {T1}$, the medial FL length $\mathrm {L}_\mathrm {2}$, and the FL diameter $\mathrm {D}_\mathrm {FL}$, see Fig. 3. Larger tears and a more proximal entry tear together with smaller FL diameter and medial FL lengths (apart from extreme values) increase the risk of FL patency. Investigating these morphological conditions revealed that they are prone to higher shear rates in the FL, which prevents the FL to thrombose. The importance of the medial FL length $\mathrm {L}_\mathrm {2}$ was also reported in numerical studies of Menichini and Xu (2016). Furthermore, Evangelista et al. (2012) demonstrated the importance of the size of the proximal tear and its location in predicting a patent FL and causing undesirable outcomes. Also, several studies have reported an influence of the tear size and location on the hemodynamics and the correlation to adverse outcomes (Mohr-Kahaly et al. 1989; Iwai et al. 1991; Tsai et al. 2008; Qing et al. 2012; Chen et al. 2013; Rudenick et al. 2013; Canchi et al. 2018; Salameh et al. 2019; Zadrazil et al. 2020; Chung et al. 2000a, b). Due to their low overall sensitivity index (Sect. 3.1), the proximal and distal FL lengths $\mathrm {L}_\mathrm {1}$ and $\mathrm {L}_\mathrm {3}$ and the TL diameter $\mathrm {D}_\mathrm {TL}$ have no significant influence on thrombus formation. Consequently, they can be considered as model constants in future studies. In summary, only five of the eight dimensionless morphological parameters induce a variation in the volume percentage of developed thrombus $\overline{\phi }_\mathrm {th,D}$. Thus, the model complexity could potentially be reduced by about 38 %.

Our results indicate (compare Fig. 4) that an increase in the proximal tear size $\mathrm {H}_\mathrm {T1}$ leads to a higher risk of having a patent FL. Similar results were reported in the study of Evangelista et al. (2012). Several studies have also shown the importance of the aortic diameter and the FL diameter in predicting adverse outcomes (Marui et al. 1999; Trimarchi et al. 2006; Song et al. 2007; Trimarchi et al. 2013; Kudo et al. 2014; Naim et al. 2016; Matsushita et al. 2018). As reported by Song et al. (2007), an initial FL diameter of $\mathrm {D}_\mathrm {FL}\ge 22$ mm is a predictor of late adverse events. Interestingly, our results likewise indicated that the $\mathrm {D}_\mathrm {FL}\ge 22$ mm increases the risk of partial thrombosis. Note however that Song et al. (2007) did not correlate adverse events and mortality rate with partial thrombosis. Trimarchi et al. (2013) found that patients with partially thrombosed FL often have an initially larger aortic diameter. Figure 4 indicates consistent results in the sense that the TL diameter $\mathrm {D}_\mathrm {TL}$ has no noticeable influence. However, the FL diameter $\mathrm {D}_\mathrm {FL}$ has a significant influence on increasing the risk of partially thrombosed FL. Also in the study by Trimarchi et al. (2013) it is pointed out that a larger aortic diameter increases the possibility of thrombus formation in the FL, which agrees with our findings, since the risk of patency decreases with a larger FL diameter $\mathrm {D}_\mathrm {FL}$. Trimiarchi and co-authors also mentioned that there is no clear explanation for this. In the current study, we have shown that it is not possible to understand the complex process of thrombus formation by examining only one specific parameter. There is a combination of morphological parameters that determine the prospect of FL thrombosis.

Dimensionless parameters were introduced to consider variations between patients and provide more general information to improve future decision-making. In Fig. 5, we have shown that several parameters influence the degree of thrombosis in the FL. This is discussed further below. The critical value of the FL-to-TL diameter ratio $\mathrm {D}_\mathrm {FL}^*=1$ plays an evident role. Increasing the FL-to-TL diameter ratio $\mathrm {D}_\mathrm {FL}^*$ up to 1 reduces the risk of having a patent FL by about 20 %. At the same time, the probability of developing a completely thrombosed FL increases by a similar amount, while the risk of a partially thrombosed FL remains equally likely. But, if $\mathrm {D}_\mathrm {FL}^*\ge 1$, the risk of partial thrombosis rises up to about 50 %, the likelihood of a completely thrombosed FL decreases by circa 20 %, and the probability of having a patent FL continuously decreases. No risk of FL patency is found in morphologies where the FL diameter is much larger than the TL diameter, i.e. $\mathrm {D}_\mathrm {FL}^*\ge 2.5$, but the risk of partial thrombosis is elevated. Larger FL diameters contribute to the hemodynamic conditions in such a way that they reduce the total shear rate and shear stress. In other words, there are more areas in the FL that favor thrombus formation. Furthermore, the finding that the TL diameter $\mathrm {D}_\mathrm {TL}$ is not a sensitive parameter and shows no significant influence on the probability variations agrees with the results of Trimarchi et al. (2013). Matsushita et al. (2018) reported that an FL diameter larger than TL diameter is a significant risk factor for serious adverse events in TBAD. Based on the presented results, we may speculate that the finding of Matsushita et al. (2018) is related to the increased risk of partial thrombosis.

When $\mathrm {H}_\mathrm {T1}^*$ is close to 0, the proximal tear is very slightly open. As a result, the FL volumetric flow rate decreases and the local hemodynamic condition tends toward a more likely complete thrombosis and no risk of patency. This result agrees with Evangelista et al. (2012). Higher values of the distal to proximal tear ratio $\mathrm {H}_\mathrm {T2}^*$ correspond to the condition that the proximal tear is much smaller than the distal tear. This situation favors complete thrombosis rather than patency, such that there is no measured patent FL for $\mathrm {H}_\mathrm {T2}^*\ge 8$. Our investigations showed that an increase in $\mathrm {H}_\mathrm {T2}^*$ leads to a higher chance of proximal thrombus formation, which is attributed to the low flow exchange near the entry tear. This also results in a lower overall probability of patency. By contrast, lower values of the distal to proximal tear ratio $\mathrm {H}_\mathrm {T2}^*$ increase the risk of FL patency. Recent research by Fleischmann and Burris (Fleischmann and Burris 2021) showed that an entry tear 1.2 cm$^{2}$ larger than the exit tear increases the rate of adverse events. In our case, such a configuration corresponds to $\mathrm {H}_\mathrm {T2}^*\le 1$, with the risk of patent FL increasing and the probability of complete thrombosed FL decreasing. Both scenarios are known to cause undesirable events (Bernard et al. 2001; Akutsu et al. 2004; Sueyoshi et al. 2004; Trimarchi et al. 2006; Tsai et al. 2007; Fattouch et al. 2009; Evangelista et al. 2012; Trimarchi et al. 2013).

The proximal tear deviation $\mathrm {x}_\mathrm {T1}^*$ has a significant impact on increasing the risk of patency and reducing the occurrence of a completely thrombosed FL. When increasing the proximal tear deviation from $\mathrm {x}_\mathrm {T1}^*= 0$, a rapid increase in the probability can be observed until the FL patency probability reaches about 50 %. In addition, complete thrombosis does not occur. Evangelista et al. (2012) indicated a similar influence on FL patency. However, we found no impact of the proximal tear deviation $\mathrm {x}_\mathrm {T1}^*$ on a partially thrombosed FL. The medial slenderness $\mathrm {L}_\mathrm {2}^*$ shows a significant effect on the three stages of FL thrombosis. At $\mathrm {L}_\mathrm {2}^*={10}$ the probability of a complete thrombosis is maximal and at the same time the risk of a partial thrombosis is zero. The risk of FL patency increases from there, while the certainty of complete thrombosis diminishes.

Finally, larger values of proximal and distal slenderness $\mathrm {L}_\mathrm {1}^*$ and $\mathrm {L}_\mathrm {3}^*$ increase the risk of partial thrombosis. At levels above 3.5 in particular, the risk is elevated. In addition, the probability of complete thrombosis decreases to $\mathrm {L}_\mathrm {1}^*=\mathrm {L}_\mathrm {3}^*=3.5$ and the risk of FL patency increases to $\mathrm {L}_\mathrm {3}^*=3.5$.

With regard to clinical applicability, this study suggests that by extracting the important morphological parameters from computed tomography scans of the patients, clinicians might predict FL thrombosis in TBAD and identify the patients at risk of insufficient FL thrombosis. Such predictions could assist clinicians in choosing individualized medical treatments and surgical interventions. Measuring a patient’s morphological characteristics is less challenging nowadays, thanks to the constantly improving technologies of computed tomography and magnetic resonance imaging scans. However, the measured absolute dimensions of the aorta are not transferable among patients. The presented study linked the non-dimensional morphological parameters to the FL thrombosis status. Introducing dimensionless parameters brings valuable insight into addressing the extensive morphological variability of the human body.

## Limitations

A key limitation of this study is the use of idealized 2D models. This does not allow for accurately representing and simulating the complex blood flow in TBAD. Here, the blood flow is characterized by extreme flow disturbances identified by secondary motions, spiral vortexes, and chaotic recirculations. Some critical features, like the spiral helicity of the FL, are excluded in the idealized models. Considering that the thrombus formation in TBAD is driven by hemodynamics, which is directly driven by morphology, future work should include the simulation of blood flow in more realistic TBAD morphologies, in order to improve the clinical relevance.

Moreover, the interaction between the blood flow and the mechanics of the aortic wall are neglected to reduce the computational effort. Chong et al. (2022) considered the aortic wall compliance and flap motion to predict the FL thrombosis in a 3D idealized geometry of TBAD. In that study, the required time for each simulation was approximately two to three months. Chong et al. (2022) concluded that accounting for the interaction of blood flow with the flap and the wall increases the amount of thrombus in the FL by 25 %. However, in previous studies of Menichini et al. (2016), Menichini et al. (2018), Armour et al. (2020), and Armour et al. (2022), as well as in a recent study by Jafarinia et al. (2022) it was found that it is possible to predict the FL thrombosis using a rigid wall assumption for patient-specific cases. To validate the results, clinical research is needed to understand the role of morphology in predicting FL thrombosis.

Nevertheless, we found that many conclusions from the current study coincide with the findings in the literature. Besides being a proof of concept for the systematic use of computer simulations in TBAD, we were also able to identify several parameters with little influence on FL thrombosis. In another study, we found that the model of thrombus formation can be further simplified (Melito et al. 2020). With such a simplified model and a reduced set of parameters, a similar 3D study would appear to be possible in the foreseeable future. For such future applications, a virtual cohort could be used for the simulations. For this purpose, a database with computed tomography scans could be utilized to generate virtual cohorts which would allow simulations to be performed that capture the variability between patients.

## Conclusion

Clinical decision-making is often based on categorizing low- and high-risk patients based on various criteria. In TBAD, the degree of thrombosis in the FL plays a dominant role in classifying patients at risk. As previously outlined, clinical studies have shown that the morphology and the degree of thrombus formation are connected to adverse events in TBAD. However, controversial and even conflicting results were reported. This study presented a computational model to identify the major morphological parameters affecting FL thrombosis using a global sensitivity analysis. Therefore, we related the extent of thrombus formation to morphology. We then showed that the most sensitive parameters affecting FL thrombosis are FL diameter, medial FL length, and size and location of tears. Taking into account non-dimensionalization of morphology, we computed the probability of having a patent, a partially, and a completely thrombosed FL to identify risk groups. We derived a function using the morphological parameters as input and computed the degree of thrombosis to classify the morphologies into patent FL, partially and completely thrombosed FL. Although we used simplified morphologies of the dissected aorta in the current study, it is encouraging to see that the results agree well with the available clinical studies. We hope that our findings will complement clinical research by providing new insights into the relationship between TBAD morphologies and thrombus formation.

## Supplementary Information

Below is the link to the electronic supplementary material.

Supplementary file 1 (xlsx 468 KB)

## References

[CR1] Akutsu K, Nejima J, Kiuchi K, Sasaki K, Ochi M, Tanaka K, Takano T (2004). Effects of the patent false lumen on the long-term outcome of type B acute aortic dissection. Eur J Cardiothorac Surg.

[CR2] Alastruey J, Xiao N, Fok H, Schaeffter T, Figueroa CA (2016). On the impact of modelling assumptions in multi-scale, subject-specific models of aortic haemodynamics. J R Soc Interface.

[CR3] Alexanderian A, Gremaud PA, Smith RC (2020). Variance-based sensitivity analysis for time-dependent processes. Reliab Eng Syst Saf.

[CR4] Armour CH, Menichini C, Milinis K, Gibbs RGJ, Xu XY (2020). Location of reentry tears affects false lumen thrombosis in aortic dissection following TEVAR. J Endovasc Ther.

[CR5] Armour CH, Menichini C, Hanna L, Gibbs RGJ, Xu XY (2022) Computational modeling of flow and thrombus formation in type B aortic dissection: the influence of False Lumen perfused side branches. In Solid (Bio)mechanics: Challenges of the Next Decade. Studies in Mechanobiology, Tissue Engineering and Biomaterials, chapter 2. Springer, Cham. pp 53–72 10.1007/978-3-030-92339-6_2

[CR6] Ben Ahmed S, Dillon-Murphy D, Figueroa CA (2016). Computational study of anatomical risk factors in idealized models of type B aortic dissection. Eur J Vasc Endovasc Surg.

[CR7] Bernard Y, Zimmermann H, Chocron S, Litzler JF, Kastler B, Etievent JP, Meneveau N, Schiele F, Bassand JP (2001). False lumen patency as a predictor of late outcome in aortic dissection. Am J Cardiol.

[CR8] Blatman G, Sudret B (2010). An adaptive algorithm to build up sparse polynomial chaos expansions for stochastic finite element analysis. Probabilistic Eng Mech.

[CR9] Blatman G, Sudret B (2011). Adaptive sparse polynomial chaos expansion based on least angle regression. J Comput Phys.

[CR10] Boudoulas KD, Triposkiadis F, Stefanadis C, Boudoulas H (2018). Aortic size and aortic dissection: does one size fit all?. Cardiology.

[CR11] Canchi S, Guo X, Phillips M, Berwick Z, Kratzberg J, Krieger J, Roeder B, Haulon S, Chambers S, Kassab GS (2018). Role of re-entry tears on the dynamics of type B dissection flap. Ann Biomed Eng.

[CR12] Carreau PJ (1968) Rheological equations from molecular network theories. PhD thesis, University of Wisconsin, Madison

[CR13] Chen D, Müller-Eschner M, von Tengg-Kobligk H, Barber D, Böckler D, Hose R, Ventikos Y (2013). A patient-specific study of type-B aortic dissection: evaluation of true-false lumen blood exchange. Biomed Eng Online.

[CR14] Chong MY, Gu B, Armour CH, Dokos S, Oong ZC, Xu XY, Lim E (2022). An integrated fluid-structure interaction and thrombosis model for type b aortic dissection. Biomech Model Mechanobiol.

[CR15] Chung J, Elkins C, Sakai T, Kato N, Vestring T, Semba C, Slonim S, Dake M (2000). True-lumen collapse in aortic dissection: part I. Evaluation of causative factors in phantoms with pulsatile flow. Radiology.

[CR16] Chung JW, Elkins C, Sakai T, Kato N, Vestring T, Semba CP, Slonim SM, Dake MD (2000). True-lumen collapse in aortic dissection: part II. Evaluation of treatment methods in phantoms with pulsatile flow. Radiology.

[CR17] Craiem D, Alsac JM, Casciaro ME, El Batti S, Mousseaux E, Sirieix ME, Simon A (2016). Association between thoracic aorta calcium and thoracic aorta geometry in a cohort of asymptomatic participants at increased cardiovascular risk. Rev Esp Cardiol.

[CR18] Erbel R (2006). Aortic dimensions and the risk of dissection. Heart.

[CR19] Erbel R, Alfonso F, Boileau C, Dirsch O, Eber B, Haverich A, Rakowski H, Struyven J, Radegran K, Sechtem U, Taylor J, Zollikofer C, Klein WW, Mulder B, Providencia LA (2001). Diagnosis and management of aortic dissection. Eur Heart J.

[CR20] Evangelista A, Salas A, Ribera A, Ferreira-González I, Cuellar H, Pineda V, González-Alujas T, Bijnens B, Permanyer-Miralda G, Garcia-Dorado D (2012). Long-term outcome of aortic dissection with patent false lumen. Circulation.

[CR21] Fan Y, Cheng S, Qing K, Chow K (2010). Endovascular repair of type B aortic dissection: a study by computational fluid dynamics. J Biomed Sci Eng.

[CR22] Fattouch K, Sampognaro R, Navarra E, Caruso M, Pisano C, Coppola G, Speziale G, Ruvolo G (2009). Long-term results after repair of type A acute aortic dissection according to false lumen patency. Ann Thorac Surg.

[CR23] Fleischmann D, Burris N (2021). Entry tear dominance at CT angiography predicts long-term clinical outcomes in aortic dissection: another piece of the puzzle. Radiol Cardiothorac Imaging.

[CR24] Ghanem RG, Spanos PD (1991). Stochastic finite elements: a spectral approach.

[CR25] Gott VL, Greene PS, Alejo DE, Cameron DE, Naftel DC, Miller DC, Gillinov AM, Laschinger JC, Borst HG, Cabrol CEA, Cooley DA, Coselli JS, David TE, Griepp RB, Kouchoukos NT, Turina MI, Pyeritz RE (1999). Replacement of the aortic root in patients with Marfan’s syndrome. New Eng J Med.

[CR26] Iooss B, Lemaître P (2015) A review on global sensitivity analysis methods. In Uncertainty Management in Simulation-Optimization of Complex Systems. Springer US. , pp 101–122 10.1007/978-1-4899-7547-8_5

[CR27] Iwai F, Sostman HD, Evans AJ, Nadel SN, Hedlund LW, Beam CA, Charles HC, Spritzer CE (1991). Cine phase-contrast magnetic resonance imaging for analysis of flow phenomena in experimental aortic dissection. Invest Radiol.

[CR28] Jafarinia A, Müller TS, Windberger U, Brenn G, Hochrainer T (2020). Blood rheology influence on false lumen thrombosis in type B aortic dissection. J Biosci Bioeng.

[CR29] Jafarinia A, Armour CH, Gibbs RGJ, Xu XY, Hochrainer T (2022). Shear-driven modelling of thrombus formation in type B aortic dissection. Front Bioeng Biotechnol.

[CR30] Jonker FHW, Trimarchi S, Rampoldi V, Patel HJ, O’Gara P, Peterson MD, Fattori R, Moll FL, Voehringer M, Pyeritz RE, Hutchison S, Montgomery D, Isselbacher EM, Nienaber CA, Eagle KA (2012). Aortic expansion after acute type B aortic dissection. Ann Thorac Surg.

[CR31] Karmonik C, Bismuth J, Redel T, Anaya-Ayala JE, Davies MG, Shah DJ, Lumsden AB (2010) Impact of tear location on hemodynamics in a type B aortic dissection investigated with computational fluid dynamics. In 2010 Annual international conference of the IEEE engineering in medicine and biology, pp 3138–3141. 10.1109/IEMBS.2010.5627193 10.1109/IEMBS.2010.5627193 21096590

[CR32] Karmonik C, Bismuth J, Shah DJ, Davies MG, Purdy D, Lumsden AB (2011). Computational study of haemodynamic effects of entry- and exit-tear coverage in a DeBakey type III aortic dissection: technical report. Eur J Vasc Endovasc Surg.

[CR33] Keramati H, Birgersson E, Ho JP, Kim S, Chua KJ, Leo HL (2020). The effect of the entry and re-entry size in the aortic dissection: a two-way fluid-structure interaction simulation. Biomech Model Mechanobiol.

[CR34] Khoynezhad A, Walot I, Kruse MJ, Rapae T, Donayre CE, White RA (2010). Distribution of intimomedial tears in patients with type B aortic dissection. J Vasc Surg.

[CR35] Kudo T, Mikamo A, Kurazumi H, Suzuki R, Morikage N, Hamano K (2014). Predictors of late aortic events after Stanford type B acute aortic dissection. J Thorac Cardiovasc Surg.

[CR36] Le Gratiet L, Marelli S, Sudret B (2017) Metamodel-based sensitivity analysis: polynomial chaos expansions and Gaussian processes. In: Ghanem R, Higdon D, and Owhadi H (eds), Handbook of Uncertainty Quantification, pp 1289–1325. Springer. 10.1007/978-3-319-12385-1_38

[CR37] LeMaire SA, Russell L (2011). Epidemiology of thoracic aortic dissection. Nat Rev Cardiol.

[CR38] Marelli S, Sudret B (2014) UQLab: a framework for uncertainty quantification in Matlab. In Vulnerability, Uncertainty, and Risk (ASCE), pp 2554–2563. 10.1061/9780784413609.257

[CR39] Marui A, Mochizuki T, Mitsui N, Koyama T, Kimura F, Horibe M (1999) Toward the best treatment for uncomplicated patients with type B acute aortic dissection: a consideration for sound surgical indication. Circulation, 100(suppl_2):II–275. 10.1161/01.cir.100.suppl_2.ii-275 10.1161/01.cir.100.suppl_2.ii-275 10567316

[CR40] Matsushita A, Hattori T, Tsunoda Y, Sato Y, Mihara W (2018). Impact of initial aortic diameter and false-lumen area ratio on type B aortic dissection prognosis. Interact Cardiovasc Thorac Surg.

[CR41] Melito GM, Jafarinia A, Hochrainer T, Ellermann K (2020). Sensitivity analysis of a phenomenological thrombosis model and growth rate characterisation. J Biosci Bioeng.

[CR42] Menichini C, Xu XY (2016). Mathematical modeling of thrombus formation in idealized models of aortic dissection: initial findings and potential applications. J Math Biol.

[CR43] Menichini C, Cheng Z, Gibbs RGJ, Xu XY (2016). Predicting false lumen thrombosis in patient-specific models of aortic dissection. J R Soc Interface.

[CR44] Menichini C, Cheng Z, Gibbs RGJ, Xu XY (2018). A computational model for false lumen thrombosis in type B aortic dissection following thoracic endovascular repair. J Biomech.

[CR45] Mohr-Kahaly S, Erbel R, Rennollet H, Wittlich N, Drexler M, Oelert H, Meyer J (1989). Ambulatory follow-up of aortic dissection by transesophageal two-dimensional and color-coded doppler echocardiography. Circulation.

[CR46] Naim WNWA, Ganesan PB, Sun Z, Liew YM, Qian Y, Lee CJ, Jansen S, Hashim SA, Lim E (2016). Prediction of thrombus formation using vortical structures presentation in Stanford type B aortic dissection: a preliminary study using CFD approach. Appl Math Model.

[CR47] Nienaber CA, Clough R, Sakalihasan N, Suzuki T, Gibbs R, Mussa F, Jenkins M, Thompson M, Evangelista A, Yeh J, Cheshire N, Rosendahl U, Pepper J (2016). Aortic dissection. Nat Rev Dis Primers.

[CR48] Pape LA, Tsai TT, Isselbacher EM, Oh JK, O’Gara PT, Evangelista A, Fattori R, Meinhardt G, Trimarchi S, Bossone E, Suzuki T, Cooper JV, Froehlich JB, Nienaber CA, Eagle KA (2007). Aortic diameter $$\ge 5.5$$ cm is not a good predictor of type A aortic dissection. Circulation.

[CR49] Pepe A, Li J, Rolf-Pissarczyk M, Gsaxner C, Chen X, Holzapfel GA, Egger J (2020). Detection, segmentation, simulation and visualization of aortic dissections: a review. Med Image Anal.

[CR50] Qing KX, Chan YC, Lau SF, Yiu WK, Ting AC, Cheng SW (2012). Ex-vivo haemodynamic models for the study of Stanford type B aortic dissection in isolated porcine aorta. Eur J Vasc Endovasc Surg.

[CR51] Quint LE, Platt JF, Sonnad SS, Deeb GM, Williams DM (2003). Aortic intimal tears: detection with spiral computed tomography. J Endovasc Ther.

[CR52] Rudenick PA, Bijnens BH, García-Dorado D, Evangelista A (2013). An in vitro phantom study on the influence of tear size and configuration on the hemodynamics of the lumina in chronic type B aortic dissections. J Vasc Surg.

[CR53] Rudenick PA, Segers P, Pineda V, Cuellar H, García-Dorado D, Evangelista A, Bijnens BH (2017) False lumen flow patterns and their relation with morphological and biomechanical characteristics of chronic aortic dissections. computational model compared with magnetic resonance imaging measurements. PLoS One, 12(1):e0170888. 10.1371/journal.pone.0170888 10.1371/journal.pone.0170888 PMC5270334 28125720

[CR54] Sailer AM, Van Kuijk SMJ, Nelemans PJ, Chin AS, Kino A, Huininga M, Schmidt J, Mistelbauer G, Bäumler K, Chiu P (2017). Computed tomography imaging features in acute uncomplicated Stanford type-B aortic dissection predict late adverse events. Circ Cardiovasc Imaging.

[CR55] Salameh E, Saade C, Oweis GF (2019). Experimental insight into the hemodynamics and perfusion of radiological contrast in patent and non-patent aortic dissection models. Cardiovasc Eng Technol.

[CR56] Saltelli A (2002). Making best use of model evaluations to compute sensitivity indices. Comput Phys Commun.

[CR57] Saltelli A, Ratto M, Andres T, Campolongo F, Cariboni J, Gatelli D, Saisana M, Tarantola S (2008). Global sensitivity analysis: the primer.

[CR58] Sobol’ IM (1990). On sensitivity estimation for nonlinear mathematical models. Math Comput Simul.

[CR59] Sobol’ IM (2001). Global sensitivity indices for nonlinear mathematical models and their Monte Carlo estimates. Math Comput Simul.

[CR60] Song JM, Kim SD, Kim JH, Kim MJ, Kang DH, Seo JB, Lim TH, Lee JW, Song MG, Song JK (2007). Long-term predictors of descending aorta aneurysmal change in patients with aortic dissection. J Am Coll Cardiol.

[CR61] Spinelli D, Benedetto F, Donato R, Piffaretti G, Marrocco-Trischitta MM, Patel HJ, Eagle KA, Trimarchi S (2018). Current evidence in predictors of aortic growth and events in acute type B aortic dissection. J Vasc Surg Cases.

[CR62] Strotzer M, Aebert H, Lenhart M, Nitz W, Wild T, Manke C, Volk M, Feuerbach S (2000). Morphology and hemodynamics in dissection of the descending aorta. Assessment with MR imaging. Acta Radiol.

[CR63] Sudret B (2008). Global sensitivity analysis using polynomial chaos expansions. Reliab Eng Syst Saf.

[CR64] Sueyoshi E, Sakamoto I, Hayashi K, Yamaguchi T, Imada T (2004) Growth rate of aortic diameter in patients with type B aortic dissection during the chronic phase. Circulation, 110(11_suppl_1):II256–61. 10.1161/01.CIR.0000138386.48852.b6 10.1161/01.CIR.0000138386.48852.b6 15364872

[CR65] Sueyoshi E, Sakamoto I, Uetani M (2009). Growth rate of affected aorta in patients with type B partially closed aortic dissection. Ann Thorac Surg.

[CR66] Tanaka A, Sakakibara M, Ishii H, Hayashida R, Jinno Y, Okumura S, Okada K, Murohara T (2014). Influence of the false lumen status on clinical outcomes in patients with acute type B aortic dissection. J Vasc Surg.

[CR67] Tang B (1993). Orthogonal array-based Latin hypercubes. J Am Stat Assoc.

[CR68] Trimarchi S, Nienaber CA, Rampoldi V, Myrmel T, Suzuki T, Bossone E, Tolva V, Deeb MG, Upchurch GR, Jr Cooper JV, Fang J, Isselbacher EM, Sundt TMr, Eagle KA (2006) Role and results of surgery in acute type B aortic dissection: insights from the International Registry of Acute Aortic Dissection (IRAD). Circulation, 114:I357–64. 10.1161/CIRCULATIONAHA.105.000620 10.1161/CIRCULATIONAHA.105.000620 16820600

[CR69] Trimarchi S, Tolenaar JL, Jonker FHW, Murray B, Tsai TT, Eagle KA, Rampoldi V, Verhagen HJM, van Herwaarden JA, Moll FL, Muhs BE, Elefteriades JA (2013). Importance of false lumen thrombosis in type B aortic dissection prognosis. J Thorac Cardiovasc Surg.

[CR70] Tsai TT, Evangelista A, Nienaber CA, Myrmel T, Meinhardt G, Cooper JV, Smith DE, Suzuki T, Fattori R, Llovet A, Froehlich J, Hutchison S, Distante A, Sundt T, Beckman J, Januzzi JL, Isselbacher EM, Eagle KA (2007). Partial thrombosis of the false lumen in patients with acute type B aortic dissection. N Engl J Med.

[CR71] Tsai TT, Schlicht MS, Khanafer K, Bull JL, Valassis DT, Williams DM, Berguer R, Eagle KA (2008). Tear size and location impacts false lumen pressure in an ex vivo model of chronic type B aortic dissection. J Vasc Surg.

[CR72] Vasava P, Jalali P, Dabagh M, Kolari PJ (2012). Finite element modelling of pulsatile blood flow in idealized model of human aortic arch: study of hypotension and hypertension. Comput Math Methods Med.

[CR73] Weller HG, Tabor G, Jasak H, Fureby C (1998). A tensorial approach to computational continuum mechanics using object-oriented techniques. Comput Phys.

[CR74] Westerhof N, Lankhaar JW, Westerhof BE (2008). The arterial Windkessel. Med Biol Eng Comput.

[CR75] Wiener N (1938). The homogeneous chaos. Am J Math.

[CR76] Xiu D (2010). Numerical methods for stochastic computations.

[CR77] Xiu D, Karniadakis GE (2002). Modeling uncertainty in steady state diffusion problems via generalized polynomial chaos. Comput Methods Appl Mech Eng.

[CR78] Xiu D, Karniadakis GE (2005). The Wiener-Askey polynomial chaos for stochastic differential equations. SIAM J Sci Comput.

[CR79] Zadrazil I, Corzo C, Voulgaropoulos V, Markides CN, Xu XY (2020). A combined experimental and computational study of the flow characteristics in a type B aortic dissection: effect of primary and secondary tear size. Chem Eng Res Des.

